# Impact of Halogen Termination and Chain Length on π-Electron Conjugation and Vibrational Properties of Halogen-Terminated Polyynes

**DOI:** 10.1021/acs.jpca.3c07915

**Published:** 2024-03-20

**Authors:** Simone Melesi, Pietro Marabotti, Alberto Milani, Bartłomiej Pigulski, Nurbey Gulia, Piotr Pińkowski, Sławomir Szafert, Mirella Del Zoppo, Chiara Castiglioni, Carlo S. Casari

**Affiliations:** † Department of Energy, Micro and Nanostructured Materials Laboratory - NanoLab, Energy, Politecnico di Milano, Via Ponzio 34/3, Milano 20133, Italy; ‡ Faculty of Chemistry, University of Wrocław, 14 F. Joliot-Curie, Wrocław 50-383, Poland; § Department of Chemistry, Materials and Chemical Engineering “Giulio Natta”, Politecnico di Milano, Piazza Leonardo da Vinci 32, Milano 20133, Italy; ∥ Institut für Physik and IRIS Adlershof, Humboldt Universität zu Berlin, 12489 Berlin, Germany

## Abstract

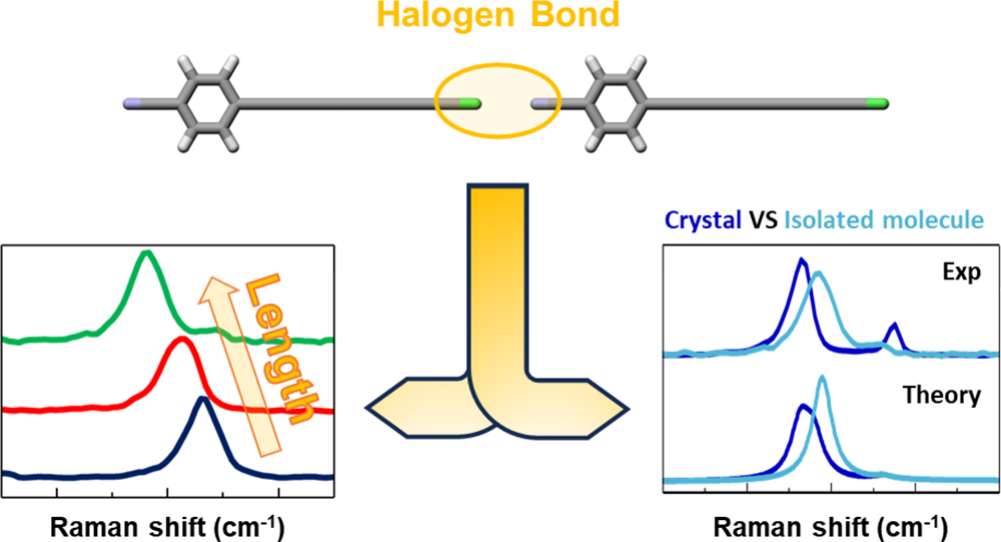

We explored the optoelectronic and vibrational properties of a new class of halogen-terminated carbon atomic wires in the form of polyynes using UV–vis, infrared absorption, Raman spectroscopy, X-ray single-crystal diffraction, and DFT calculations. These polyynes terminate on one side with a cyanophenyl group and on the other side, with a halogen atom X (X = Cl, Br, I). We focus on the effect of different halogen terminations and increasing lengths (i.e., 4, 6, and 8 sp-carbon atoms) on the π-electron conjugation and the electronic structure of these systems. The variation in the sp-carbon chain length is more effective in tuning these features than changing the halogen end group, which instead leads to a variety of solid-state architectures. Shifts between the vibrational frequencies of samples in crystalline powders and in solution reflect intermolecular interactions. In particular, the presence of head-to-tail dimers in the crystals is responsible for the modulation of the charge density associated with the π-electron system, and this phenomenon is particularly important when strong I··· N halogen bonds occur.

## Introduction

Many different carbon allotropes have been observed in nature and, in the last decades, have attracted a growing interest in material science.^1^ Graphite and diamonds represent the sp^2^ and sp^3^ carbon allotropes, respectively, but carbon atoms can also adopt a sp hybridization and form linear chains or atomic wires, featuring a strong π-electron conjugation. In the limit of an infinite number of C atoms, the linear carbon chain is an ideal 1D crystal, called carbyne. Carbyne has two possible isomers with distinct properties: cumulene, with equalized double bonds and metallic behavior, and polyyne, consisting of alternating single and triple bonds with semiconducting behavior.^2,3^ These elusive systems with infinite length are of great interest because, as suggested by the theoretical predictions, they should possess properties among the best ever recorded, like the highest Young modulus and stiffness,^4^ exceptional electron mobility in the case of cumulene,^5^ and thermal conductivity.^6^ So far, only sp-carbon chains with rather short lengths have been synthesized, with two remarkable exceptions. Linear carbon chains encapsulated inside carbon nanotubes have been obtained, showing lengths approaching the carbyne limit.^7^ Recently, the synthesis and characterization of monodispersed isolated polyynes, featuring 68 sp-carbon atoms, was reported in Patrick et al.^8^ These exceptionally long polyynes are stabilized by supramolecular encapsulation by threading them through macrocycles, and their electronic properties converge with those of carbyne.

In the case of finite size sp-carbon chains, the peculiar physical properties related to the π-electron system delocalized along a linear backbone result in important applications in photovoltaics and hydrogen storage,^9,10^ electronics,^9,11−13^ nonlinear optical (NLO) devices,^14−21^ live-cell imaging,^22^ and in other nanotechnology fields.^12,23^ Differences in the end groups or length affect the optoelectronic and vibrational properties of sp-carbon chains,^2,3,24^ making them appealing candidates for developing innovative functional materials with tunable properties.

Despite these remarkable properties, the application of sp-carbon chains is limited due to their poor stability. Indeed, they suffer from ozonolysis processes, are sensitive to heating and light irradiation, and can undergo cross-linking reactions between adjacent chains, leading to a rearrangement into more stable sp^2^ structures.^25−27^ Among different adopted strategies to stabilize these compounds, the chemical synthesis of sp-carbon chains with bulky terminations is one of the most investigated routes,^28−32^ producing up to gram-scale stable solid-state samples of sp-carbon chains.^8,30,32−41^

Among the most assessed characterization techniques, UV–vis absorption spectra of these systems present a sequence of vibronic peaks whose position is strictly dependent on their length and their terminations.^15,37,42−44^ Raman spectra of polyynes present a very characteristic mode called effective conjugation coordinate (or α) (i.e., ECC), which consists of a collective vibration of all of the CC bonds of the sp-chain. The ECC mode fits in a frequency region (1800–2300 cm^–1^) where all the other carbon nanostructures do not have any Raman-active mode, thus making the ECC band a perfect marker to detect the presence of sp-hybridized carbon structures.^24,45−47^ The frequency of the ECC band shifts with the structure of the chains, providing information on the length, terminations, π-electron conjugation, and electron–phonon coupling.^24,28,48,49^ In centrosymmetric linear sp-carbon wires, the ECC mode does not induce a variation in the molecular dipole moment and is not IR active. Nevertheless, the ECC mode becomes IR active for heteroterminations or deviations from chain linearity breaking the inversion symmetry.^50−52^

Among the possible end groups, halogen-terminated polyynes (or halopolyynes) exhibit selective reactivity and are ideal precursors to functionalize polyynes with amine,^41,53,54^ pyrrole,^55,56^ and metallorganic^57−59^ end-capping or to produce fluorescent dyes.^60^ However, only a few studies on halopolyynes are available,^61−63^ and a thorough investigation of the chemical-physical properties and potentialities of these systems is lacking. Further analysis of the role of halogen terminations and intermolecular interactions can reveal intriguing optoelectronic and conjugation properties suitable for different technological fields.

In this work, we investigated the vibrational, and optical properties, and solid-state structures of a series of 1-halopolyynes with different lengths (4, 6, and 8 carbon atoms) and halogen terminations (X = Cl, Br, and I).^64^ These systems present a cyanophenyl group as the other termination, which enhances their stability and acts as an electron-withdrawing unit.^21^ The negatively charged CN group and the terminal C–X group exhibiting electron-donor character generate a permanent dipole moment parallel to the sp-carbon backbone. These polar end groups also affect the intermolecular interactions in the solid state. The packing motif observed in the crystals results from the balance of van der Waals interactions between H atoms of the phenyl group and the conjugated π-electrons and from the electrostatic interactions (e.g., halogen bonding) between halogen and the nitrogen atoms of the nearest neighbor systems.

The halogen and the cyanophenyl terminations polarize the sp-carbon chain, making their ECC mode both Raman and IR active and allowing a detailed investigation of the vibrational properties. By observing frequency shifts and intensity modulations of the ECC peaks while passing from the solid state to solution samples, we explored the intermolecular interactions pointing to the occurrence of halogen bonds. Density functional theory (DFT) simulations of Raman and IR spectra complement the analysis, providing the vibrational assignment and giving significant information about molecular geometry, dipole moment, and orbital energies. The energy of the frontier orbitals is studied by UV–vis absorption spectroscopy in solutions. Solid-state packing and its correlation with the different molecular structures and intermolecular interactions are explored through X-ray single-crystal diffraction experiments.

## Experimental Methods

**Figure 1:**
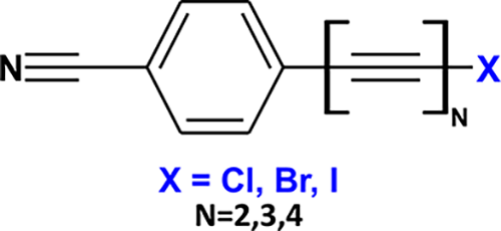
Chemical structure of 1-halopolyynes. In this picture, X represents the halogen termination (Cl, Br, and I) and N represents the number of triple bonds in the sp-carbon chain (2, 3, and 4).

The chemical structures of 1-halopolyynes are sketched in Figure 1. Each chain is identified by the label C_*n*_X, where *n* indicates the specific number of sp-carbon atoms in the polyyne chain (C_4_, C_6_, and C_8_, respectively), while X marks the different halogen terminations (Cl, Br, and I). These systems were synthesized following the method described in our previous reports.^57,58,65^ C_6_Cl and C_8_Br polyynes are new, and their synthesis and characterization are described in Supporting Information. The synthesis of these two polyynes is of particular importance since C_6_Cl and C_8_Br represent the first known examples in the literature of chlorine-terminated triyne and bromooctatetrayne, respectively. The stability of these molecules against aggregations by cross-linking reactions depends on their length and terminations: it increases by shortening the chain and passing from lighter to heavier halogen capping. Due to these stability issues, C_8_Cl has not been investigated experimentally, and only DFT analysis was performed on this polyyne.

### UV–Vis Spectroscopy

UV–vis absorption spectra of the 1-halopolyynes were recorded by dissolving the sample powders in dichloromethane (DCM, HPLC-grade, purity 99,8+%, contains amylene as a stabilizer, Sigma-Aldrich) or MeCN (acetonitrile, HPLC-grade, purity 99,9+%, Honeywell Research Chemicals) at various concentrations, i.e., from 10^–4^ to 10^–7^ M. Spectra were recorded at room temperature using a Shimadzu UV-1800 UV/visible scanning spectrophotometer with a detection range of 190–1100 nm. The sampling interval of the spectra was set to 0.2 nm.

### FT-Raman and FTIR Spectroscopy

FT-Raman and FTIR spectra of polyynes samples were recorded both in the form of powders and solutions in chloroform (purity 99.8+%, stabilized with amylene, Fisher Chemical) with different dilutions, from 10^–2^ to 10^–6^ M. FT-Raman spectra were recorded at room temperature using a Nicolet NXR9650 spectrometer equipped with an Nd-YVO4 solid-state laser emitting at 1064 nm. The resolution was set to 4 cm^–1^ and the spot size was 50 μm. We set 256 accumulations per spectrum to obtain a good signal-to-noise ratio. Spectra of solid-state samples were obtained directly by positioning the powders on the sample holder, while measurements of solutions required the use of NMR tubes. The power on the samples was approximately 600 mW for powders and 2 W for solutions.

FTIR spectra of powders were measured at room temperature with a Nicolet Nexus FTIR spectrometer coupled with a Thermo-Nicolet Continuum infrared microscope and a liquid-nitrogen-cooled MCT detector. Spectra were recorded using a Diamond Anvil Cell (DAC) in transmission mode. FTIR spectra of solutions were recorded at room temperature by using a Nicolet Nexus FTIR equipped with a DTGS detector. The measurements were performed in transmission mode using a cell for liquid samples with KBr windows. We set the number of accumulations to 32 and the resolution to 4 cm^–1^ for both solid and liquid samples to obtain a good signal-to-noise ratio.

### DFT Calculations

Geometry optimization of 1-halopolyynes and the prediction of their IR and Raman spectra were performed with first-principles calculations using the GAUSSIAN09 package.^66^ All the calculations have been carried out with PBE0 as a functional and 6-311++G (d,p) as a basis set because they were previously adopted in many other works on polyynes,^67−70^ demonstrating to provide reliable predictions of their structural, electronic, and vibrational properties.^71^ Spectra of both single molecules and dimers have been computed. Calculations based on isolated molecules will be compared to solution samples, while head-to-tail dimers (HT) are used to model the most relevant intermolecular interactions occurring in a few solid-state samples, showing evidence of halogen bonding, and are compared to powder spectra. The computed spectra were scaled by a factor of 0.96 to ease the comparison to the experiments. This factor was determined by adjusting the position of the phenyl stretching peak at around 1670 cm^–1^ in calculated spectra to that at around 1600 cm^–1^ in the experiments. This peak has been selected as an internal reference since it is highly recognizable in all the spectra and its frequency is almost independent of 1-halopolyynes’ structure.

### Single-Crystal X-ray diffraction (XRD)

Single crystals of C_4_Cl, C_4_Br, C_6_Cl, and C_6_Br were obtained by the slow evaporation of their CH_2_Cl_2_/hexane solutions. Suitable crystals were selected and measured on an Xcalibur R Gemini A Ultra or a Rigaku XtaLAB Synergy-R diffractometer. The crystals were kept at 100 K during data collection. Using Olex2,^72^ the structures were solved with the olex2.solve^73^ or SHELXS^74^ structure solution programs and refined with the SHELXL^75^ refinement package using least-squares minimization. More details are in the Supporting Information (Tables S4–S7). *CrystalExplorer*(76) was used for Hirshfeld surfaces and interaction energies analysis in the crystal structures.^77^

## Results and Discussion

### Electronic Properties and Charge Distribution of 1-Halopolyynes

**Figure 2:**
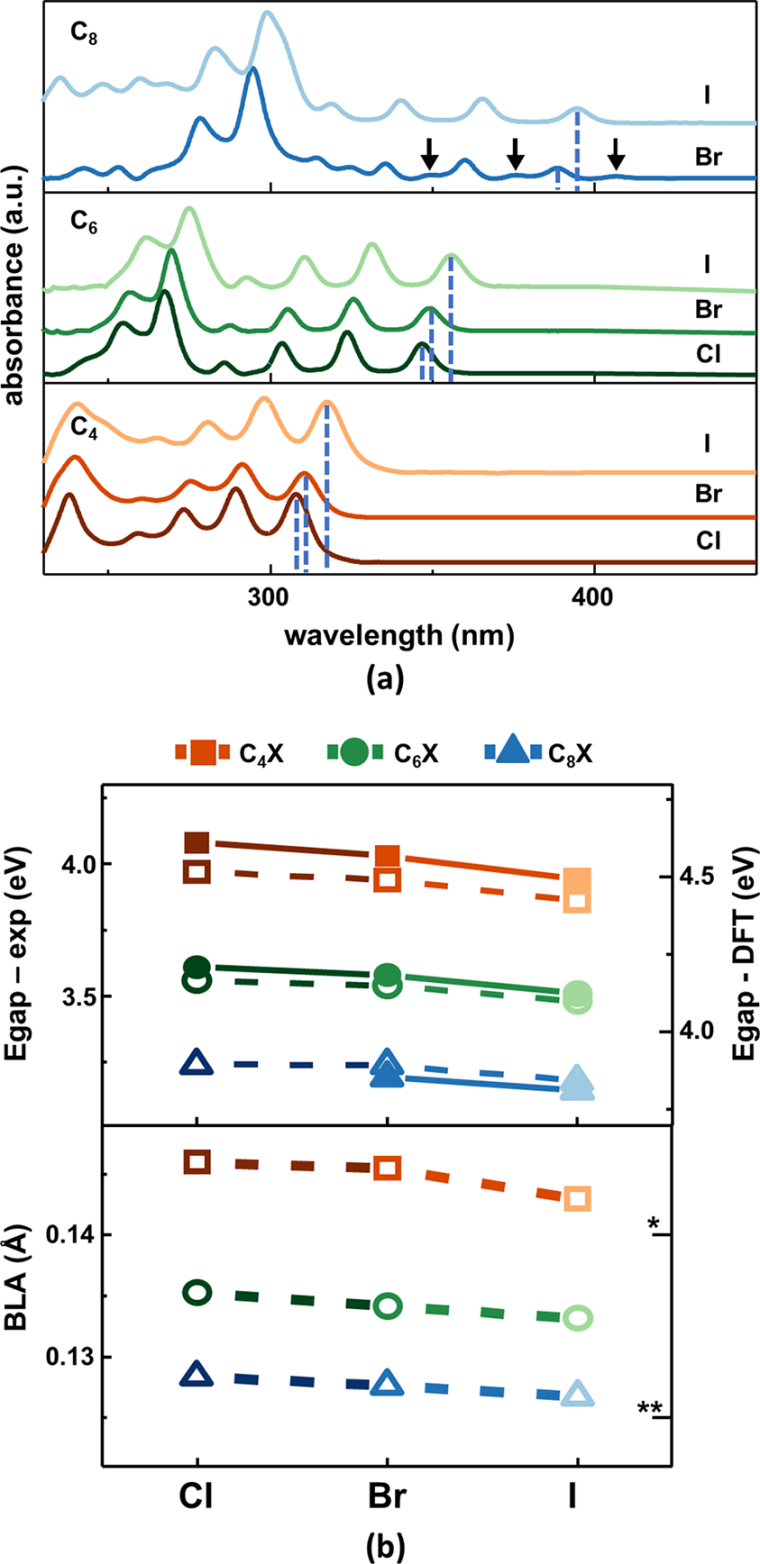
(a) UV–vis absorption spectra of the 1-halopolyynes dissolved in dichloromethane (concentration of ≈10^–5^ M). For C_8_Br, the additional vibronic sequence at lower energies is highlighted with black arrows. (b) On the top panel, the experimental optical energy gap (solid lines and full symbols), determined from the position of the highest wavelength (|0⟩_*g*_ → |0⟩_*e*_) peak of the vibronic sequence and indicated with dashed lines in the spectra of panel (a), and DFT-calculated HOMO–LUMO energy gaps (dashed lines and empty symbols) of single molecules. On the bottom panel, DFT-calculated values of the bond length alternation (BLA) parameter (dashed lines and empty symbols) of isolated molecules. As a comparison, the BLA values for hydrogen-capped (*) and amine and cyanophenyl heteroterminated (**) polyynes (with 8 sp-carbon atoms) are reported.^21,24^

Figure 2a shows the experimental UV–vis absorption spectra of 1-halopolyynes diluted in dichloromethane (concentration ≈10^–5^ M). Two distinct sequences of vibronic peaks, one at longer wavelengths composed of three absorption peaks and one at shorter wavelengths consisting of two main peaks (in C_4_X spectra only one of those peaks is distinguishable due to the proximity of dichloromethane UV–vis cutoff), are observed. Only for C_8_Br, we observed a sequence of weak peaks at lower energies (highlighted in Figure 2a with black arrows), the origin of which is not clear. It can be related to some forbidden transitions, like in the case of pyridyl end-capped oligoynes,^37^ or to the presence of impurities or products from degradation pathways, due to the lower stability compared to the other systems here investigated. Spectra at different concentrations (from 10^–4^ to 10^–7^ M, see Figure S1 in the Supporting Information) were measured, showing no difference in the position of the peaks, indicating that aggregation phenomena do not take place in this range of concentrations.

The sequences of vibronic peaks shift to longer wavelengths by increasing the number of sp-carbon atoms or passing from Cl to Br to I due to a corresponding increase in π-electron conjugation. We compared the HOMO–LUMO gap obtained by DFT calculations, with the experimental optical gap (see top panel of Figure 2b) obtained from the position (in nm) of the absorption peaks assigned to the vibronic transition from the lowest (0) vibrational level of the ground (*g*) electronic state (i.e., |0⟩_*g*_) to the lowest (0) vibrational level of the excited (*e*) electronic state (i.e., |0⟩_*e*_), namely the |0⟩_*g*_ → |0⟩_*e*_ transition. For each polyyne length, the energy gap reduces by approximately 0.1 eV from the Cl to I end group, while it decreases by 0.8 eV going from 4 to 8 sp-carbon atoms for any fixed X. DFT calculations of the HOMO–LUMO gap agree with experimental data, with a decrease of 0.08 eV from the Cl to I end groups and 0.7 eV with an increase in the chain length. The modulations induced by the change in the halogen termination are smaller than the impact of chain lengths. Similarly, the bond length alternation (BLA) of 1-halopolyynes, calculated with DFT according to Milani et al.,^24^ nicely parallels the trend of the HOMO–LUMO gap (see bottom panel in Figure 2b).

The decreasing trend of both BLA and gap values (Figure 2b) by increasing the electron-donor capability of the halogen termination (from Cl to I) is due to an increased π-electron density along the sp-carbon chain, as discussed below. However, the overall π-electron conjugation is more affected by the variations in the chain length, as already observed in previous works on polyynes with other end groups.^21,24,31^ In polyynes with 8 sp-carbon atoms, predicted BLA decreases from 0.14 Å with hydrogen terminations at both ends to 0.12 Å with amine and cyanophenyl heteroterminations.^21,24,31^ The halogenated polyynes studied in this work are placed in the middle with a theoretical BLA of about 0.13 Å.

Due to the presence of electron acceptor (CN) and electron-donor (X) terminations, these 1-halopolyynes possess a non-negligible dipole moment. DFT calculations predict a dipole moment ranging from 4.2 to 4.9 D when moving from the shortest polyyne terminated with Cl to the longest one terminated with I. The dipole moment increases with the halogen electron-donor capability (from Cl to I) of approximately 12% in C_4_X and 15% in C_8_X series, respectively (see Table S1a in the Supporting Information).

**Figure 3:**
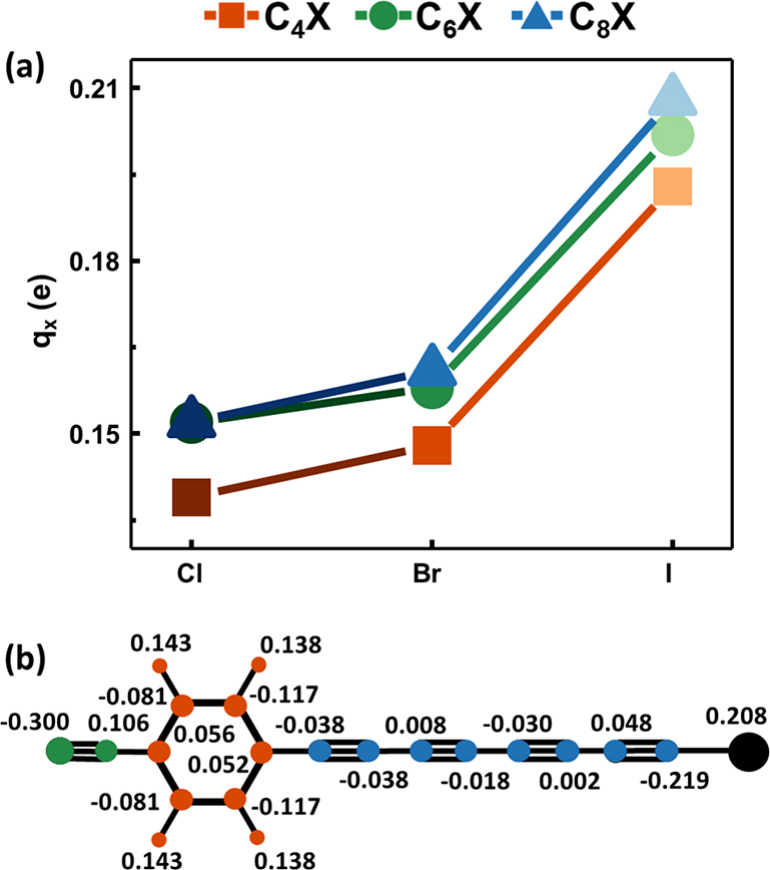
(a) IR atomic charges of the halogen atom (X) of 1-halopolyynes (C_*n*_X, X = Cl, Br, I; *n* = 4, 6, 8). IR atomic charges are obtained from the computed (DFT) atomic polar tensors.^78−80^ (b) Atomic infrared charges of C_8_I derived from the computed (DFT) atomic polar tensors. Atoms belonging to different groups are pictured with different colors (green, for the CN group, red, for the phenyl ring, blue for the sp-chain, and black for the halogen). The values are given in units of electrons (e).

The effectiveness of the polar end group in polarizing the electronic cloud of 1-halopolyynes could give rise to a “push-pull” effect^21^ resulting in a net electron charge transfer between the two ends. To analyze a possible push–pull behavior, we evaluated the charge distribution using atomic or group charges. In a previous work,^64^ charges calculated from electrostatic potentials using a grid-based method (CHELPG) allowed us to investigate the halogen bond formation in several halopolyynes, demonstrating that the halogen atoms can donate electrons to the π-system. Another effective description of the charge distribution in molecules, based on point partial charges on individual atoms (IR charges), can be obtained from DFT IR atomic polar tensors (APTs).^78−80^ The APT, P^α^ (where α labels the atoms) is a 3 × 3 tensor, which collects the three Cartesian components of the derivative of the molecular dipole moment with respect to the Cartesian displacements of the atom α, namely:  (“0” indicates the equilibrium geometry). The APT elements are also known as Born Charges and are needed, together with vibrational eigenvectors, for the calculation of the dipole derivatives with respect to the normal modes, which, in turn, determine the value of the IR absorption intensity of each mode. APTs, and thus IR charges, are available from the output of the DFT calculation of the IR spectra. The optimized geometries of C_*n*_X are planar, thus allowing to obtain partial charges for each atom α, directly from the relationship , where *z* is orthogonal to the molecular plane. As demonstrated by Dinur,^78^ this relationship provides a physically robust definition of atomic charges and, different from other theoretical models, it is fully compatible with the expression of the equilibrium molecular dipole moment in terms of point charges at the equilibrium atoms’ positions , namely . Figure 3a shows the trend of *q*_X_^0^ with the halogen (X) terminations, while Figure 3b reports all of the *q*_α_^0^ values of C_8_I, taken as a representative of 1-halopolyynes. Table S1b in the Supporting Information reports IR atomic charges of the end groups of 1-halopolyynes, i.e., nitrogen (N), halogen (X), and the C atom linked to X (C1).

All of the halogen atoms have a positive IR charge due to their capability to donate electrons to the π-electron system, which overwhelm their electron-attracting power connected to their high electronegativity (higher than that of the C atom). Moreover, the (positive) charge on iodine termination is about 35% higher than that of Cl, irrespective of the chain length (i.e., number of sp-carbon atoms *n*). Atomic charges of the halogen capping are moderately affected by *n* (as shown in Figure 3a) as the dipole moment does not increase significantly with n (the maximum variation amounts to 4% from C_4_I to C_8_I).

To provide further insights into the charge distribution in 1-halopolyynes, we calculated group charges from the sum of the IR atomic charges of a group of atoms (Table 1). The negative charge of the nitrogen atom is only partially compensated by the positive neighboring carbon atom. Indeed, the charge on the nitrile group is always negative, and its value does not vary among the investigated 1-halopolyynes. The phenyl ring is globally positive, transferring a small portion of its electronic charge to the nitrile group and the polyyne chain. The value of the ring’s charge of the various 1-halopolyynes shows a tiny increase with the chain length. The longer the chain, the larger the electronic charge withdrawn by the sp-backbone from the ring. The negative charge localized in the sp-carbon chain slightly increases with increasing length. However, the negative charge injected in the sp-carbon skeleton by the halogen atoms remains confined to the carbon atom bonded to the halogen with only a small charge transfer to the rest of the chain (Figure 3b and Table S1b in the Supporting Information). The charge withdrawing and injection by the end groups are mainly localized on the nitrile group and C–X bond, respectively. Thus, the lack of a net charge transfer between the terminations of 1-halopolyynes excludes a push–pull behavior. However, the small polarization of the chain induced by the end groups plays a significant role in determining the IR activation of the CC stretching modes and the ECC normal mode.

**Table 1:** Group Charges (Units of Electrons) of the C_*n*_X Molecules^a^

		CN	ring	sp-chain	halogen
C_4_	Cl	–0.197	0.264	–0.206	0.139
	Br	–0.196	0.265	–0.217	0.148
	I	–0.196	0.260	–0.257	0.193
C_6_	Cl	–0.195	0.268	–0.225	0.152
	Br	–0.195	0.268	–0.231	0.158
	I	–0.194	0.274	–0.285	0.205
C_8_	Cl	–0.195	0.274	–0.238	0.159
	Br	–0.194	0.273	–0.240	0.161
	I	–0.195	0.272	–0.285	0.208

a Group charges are calculated as the sum of the atomic IR charges of specific fragments of the molecules, obtained from DFT computed APTs of the isolated molecules.

### Crystal Structures and Intermolecular Interactions

**Figure 4:**
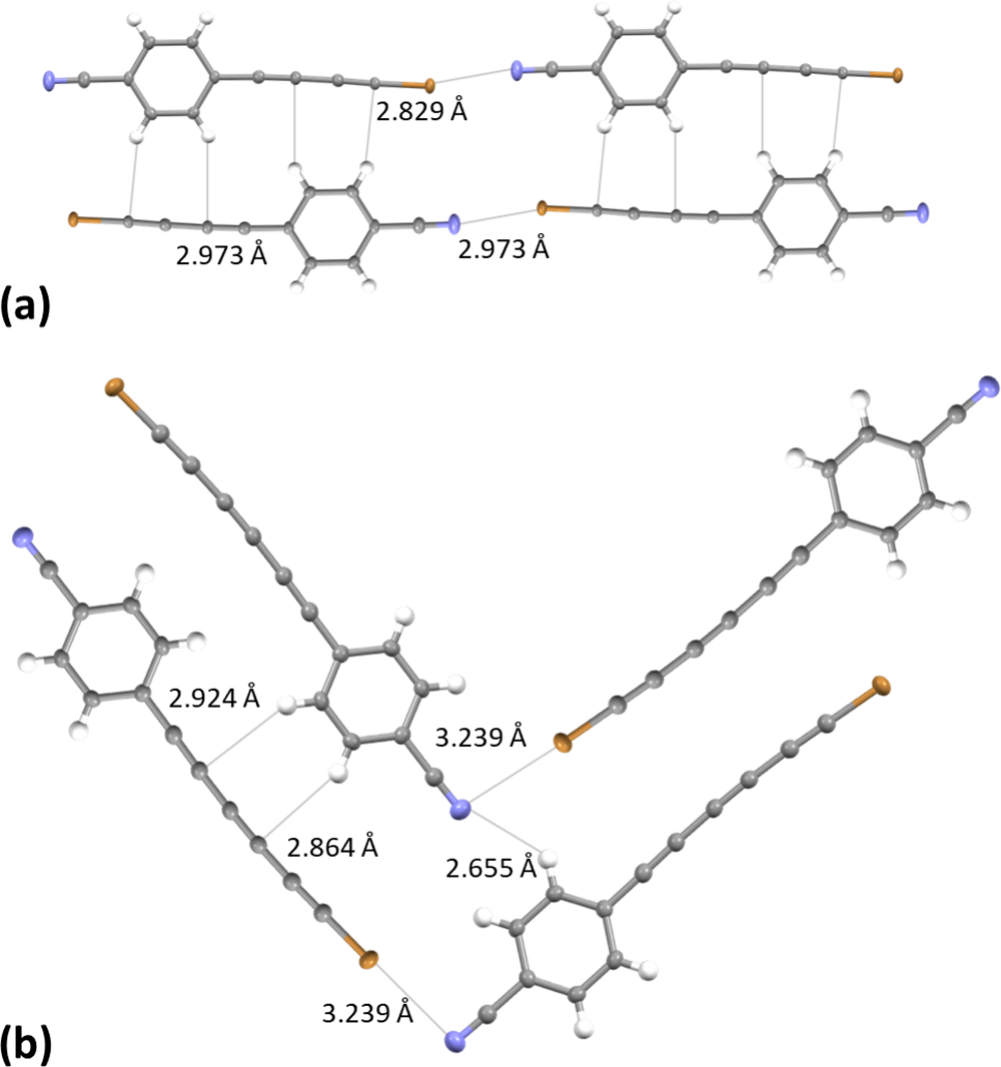
Packing motifs of 1-halopolyynes in the solid-state: (a) C_4_Br (head-to-tail) and (b) C_6_Br (herringbone-like structures). Thermal ellipsoids are given with a 50% probability.

Solid-state structures were experimentally determined using single-crystal X-ray diffraction. Single crystals suitable for measurements were obtained for all 1-halopolyynes except very unstable C_8_Br. Structures of C_*n*_I compounds were reported before,^64^ but those of C_4_Cl, C_6_Cl, C_4_Br, and C_6_Br are new and their packing motifs are presented in the Supporting Information (Figures S16–S19). The solid-state structure of C_4_Br is a typical example of the most common packing motif observed for 1-halopolyynes bearing the 4-cyanophenyl end group (Figure 4a). Usually, head-to-tail (HT) chains are formed in the crystal state due to halogen bonds. Such linear chains assemble in sheet-like structures due to C–H(ring)···π(polyyne) intermolecular interactions. However, we observed different packing motifs in two cases (C_6_Cl and C_6_Br, Figure 4b). In these systems, we do not detect any linear N···X halogen bonds leading to head-to-tail chains. As a result, nonlinear head-to-tail dimers reshape into herringbone-like structures. The reason for such a difference should be the balance between the strength of possible halogen bonds and π(polyyne/ring)···π(polyyne/ring) interactions. The decreasing value of the positive charge localized on the halogen atoms going from iodine to chlorine (see Table 1) suggests that the strength of a hypothetical halogen bond decreases from iodine to chlorine-terminated 1-halopolyynes. This behavior is proven by the data reported in Table 2, showing the shortening (Δ*R*) of the intermolecular halogen bond length, *r*(X···N) with respect to the van der Waals distance *R* = (*R*_VdW_(N) + *R*_VdW_(X)) between X and N. The increasing strength of the halogen bond from Cl to I is quantified by the increase of the Δ*R*/*R* values (from 5.8% for C_4_Cl to 18.3% for C_4_I). Among the systems forming linear dimers, C_*n*_I structures present head-to-tail chains with almost linear alignment of molecules since iodine always has the strongest halogen bond (Table 2). C_4_Cl and C_4_Br form head-to-tail chains slightly tilted with CXN angles of 152.5 and 163.8°, respectively. Interaction energy analysis (SI, Figures S20 and S21) shows that in C_*n*_Br and especially in C_*n*_Cl, energy of interactions through halogen bonding is similar to the energy of π(polyyne/ring)π(polyyne/ring) interactions between adjacent molecules. Elongating the carbon chain from butadiyne to hexatriyne, the herringbone architecture allows more favorable interactions, which are shown using Hirshfeld surface analysis (Supporting Information, Figures S22 and S23, Table S9). Surface of C···C interactions significantly increases from butadiynes (35.07 Å^2^ for C_4_Br, 41.90 Å^2^ for C_4_Cl) to hexatriynes (73.28 Å^2^ for C_6_Br, 75.13 Å^2^ for C_6_Cl). At the same time, the surface of C–H(ring)···π(polyyne) interactions slightly diminishes from about 90 Å^2^ (C_4_Cl and C_4_Br) to about 70 Å^2^ (C_6_Cl and C_6_Br). This strongly suggests that new possibilities of π···π interactions are responsible for herringbone packing motifs of C_6_Cl and C_6_Br.

**Table 2:** Intermolecular Bond Distance (*r*(X···N), in Å) of HT Dimers Compared to the Sum of the van der Waals Radii (Reported in the Supporting Information) of the Interacting Atoms *R* = (*R*_VdW_(N) + *R*_VdW_(X))^c^

compound	*r*(X···N)/Å (exp)	*r*(X···N)/Å (theo)	∠(CXN)/° (exp)	Δ*R*/*R* (exp) (%)	Δ*R*/*R* (theo) (%)
C_4_Cl^a^	3.109	3.040	152.5	5.8	7.89
C_4_Br^a^	2.973	3.012	163.8	12.5	11.41
C_4_I^b^	2.883	2.967	177.0	18.3	15.95
C_6_I^b^	2.881	2.960	178.1	18.4	16.15
C_8_I^b^	2.888	2.955	178.4	18.2	16.29

a This work.

b Known structures from Pigulski et al.^64^

c N and X label refer to nitrogen and halogen atoms, respectively. Δ*R*/*R* (%) measures the change (shortening) of the intermolecular halogen bond compared to the van der Waals distance. The sum of the van der Waals radii of the interacting atoms is reported in the SI.

Due to 1-halopolyynes’ relatively strong intermolecular interactions, such as halogen bonding, we will consider DFT models of linear head-to-tail (HT) dimers in the following discussions, which can predict some features of 1-halopolyynes crystals. As expected, C_*n*_I, for which the halogen bond is more energetic, is more affected by this intermolecular interaction than the other systems. Hence, the adopted model is limited to those polyynes that form linear structures in the crystal (i.e., C_4_X and C_*n*_I). Notwithstanding its simplicity, the dimer model can account for the main differences in the Raman and IR spectra between crystals and solutions.

**Figure 5:**
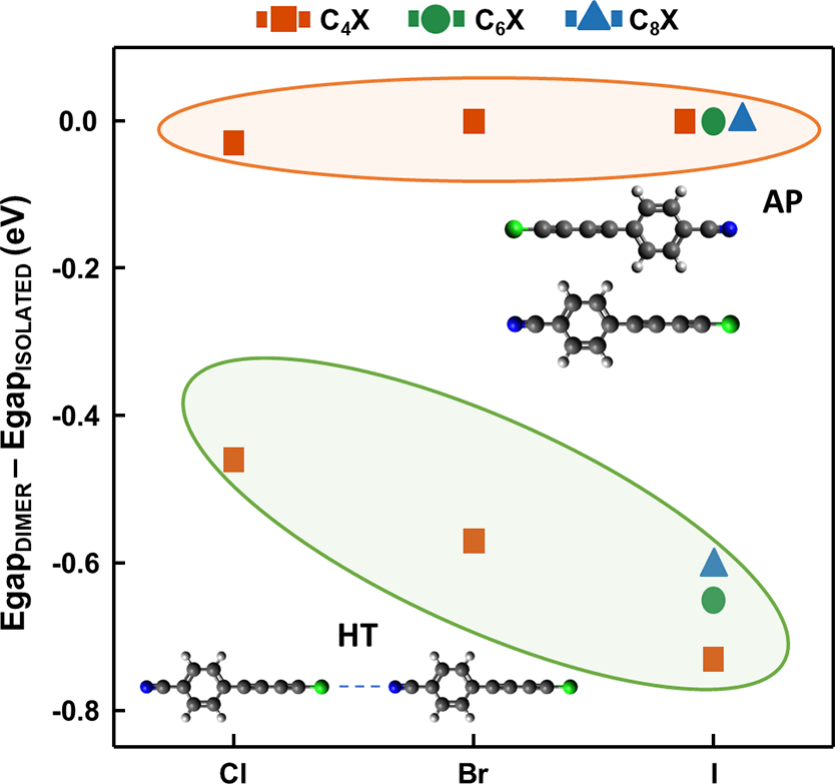
DFT calculated values of the HOMO–LUMO energy gap variation, i.e., the difference between the dimer (*E*_gapDimer_) and the single molecule (*E*_gapIsolated_) energy gaps, for head-to-tail (HT, in green circles) and antiparallel (AP, in red circles) dimers. Only C_*n*_X molecules that form linear dimers in the crystal are considered. A schematic representation of the HT and AP dimers is reported.

To assess the strength of these intermolecular interactions, we analyzed two possible dimer configurations (see their molecular structures in Figure 5): a head-to-tail (HT) configuration with a strong halogen bond, and an antiparallel (AP), coplanar, configuration, which is stabilized by C–H(ring)···π(polyyne) interactions.^64^ This comparison highlights the role of halogen bonds on relevant chemical–physical properties, such as stabilization energy, energy gap, and charge distribution of the interacting molecules.

The interaction energies of these two possible configurations have been calculated as the difference between the equilibrium energy of the dimer and twice the energies of the isolated molecules. In the case of HT dimers, DFT predicts larger interaction energies (see Table S2 in the Supporting Information) than in AP dimers, ranging from 2.5 kcal/mol in the case of C_4_Cl to 5.5 kcal/mol for C_8_I. The origin of such significant values can be ascribed to the strength of the halogen bond between the CN and halogen terminations. These interaction energies are consistent with values reported in literature^81^ for other systems able to sustain halogen bonds. In C_*n*_I, the interaction energies are only slightly affected by the chain length, while a stronger modulation occurs when changing the halogen termination, i.e., from Cl to I (see Table S2 in the Supporting Information).^82,83^

The experimental and DFT computed intermolecular bond distance, *r*(X···N) in the case of HT dimers involving C_4_X species and the series C_*n*_I are displayed in Table 2. In agreement with the experimental determinations, calculated *r*(X···N) values are smaller than the sum of the van der Waals radii of the N and X atoms, confirming that the theory can detect the presence of halogen bonds in HT dimers.

Moreover, the HT dimer gives theoretical Δ*R*/*R* values in close agreement with those of the experimental counterpart. The greatest discrepancy between theory and experiment occurs when considering C_4_Cl: its crystal shows halogen bonds that remarkably deviate from linearity, i.e., the observed XCN angle is 152.5° compared to the DFT value of 180°, which characterizes all the HT dimers. The Δ*R*/*R* value of C_4_Cl is overestimated, differently from the other 1-halopolyynes where the theory underestimates the experimental value. The linear dimer does not fully describe the C_4_Cl equilibrium structure, which may be determined by the interplay between a weak halogen bond and other intermolecular interactions.

Figure 5 shows the variation of the calculated HOMO–LUMO gap between the HT dimer and the isolated molecule (*E*_gapHT_ – *E*_gapIsolated_). This difference increases passing from Cl to I and, to a lesser extent, decreases with the chain length. The gap variation for all the HT dimers is negative; i.e., the optical gap shifts toward the visible, thus indicating an increase of the π-electron conjugation. In HT dimers, the π-electron clouds of the two polyynes are affected by the intermolecular charge transfer between the donor (X) and acceptor (cyanophenyl group) parts. In particular, the electronic charge distribution of the interacting X···N atoms is significantly perturbed, as evidenced by the variation of their atomic charges (see Table S1c in the Supporting Information). The amount of charge transferred increases according to the strength of the halogen bond (i.e., from Cl to I). The largest net charge transfer, of about 0.09 e, occurs for the C_6_I and C_8_I dimers, according to DFT calculations. This effect will play a role in determining the variations of the Raman intensities from solutions to the solid-state, as we will discuss in the following section.

AP dimers (see the molecular structure in Figure 5) feature halved interaction energy values (from 1.7 kcal/mol in the case of C_4_Cl to 3.0 kcal/mol for C_8_I) compared to those of HT dimers (see Table S2 in the Supporting Information). Figure 5 shows that the HOMO–LUMO gap of AP dimers remains almost equal to that of the isolated molecules (*E*_gapAP_ – *E*_gapIsolated_ ≈ 0). This is a consequence of the low interaction energy of AP dimers and is specifically related to the nature of this interaction, which could be described as a “through-space” interaction, not affecting the charge distribution of the two interacting 1-halopolyynes.

In the following, we will adopt the HT dimer as a reasonable model for discussing the modulation of the spectroscopic response due to intermolecular interactions occurring in solid-state samples where halogen bonding occurs.

### Vibrational Analysis of Solution and Solid-State 1-Halopolyynes: ECC and Ring Stretching Modes

**Figure 6:**
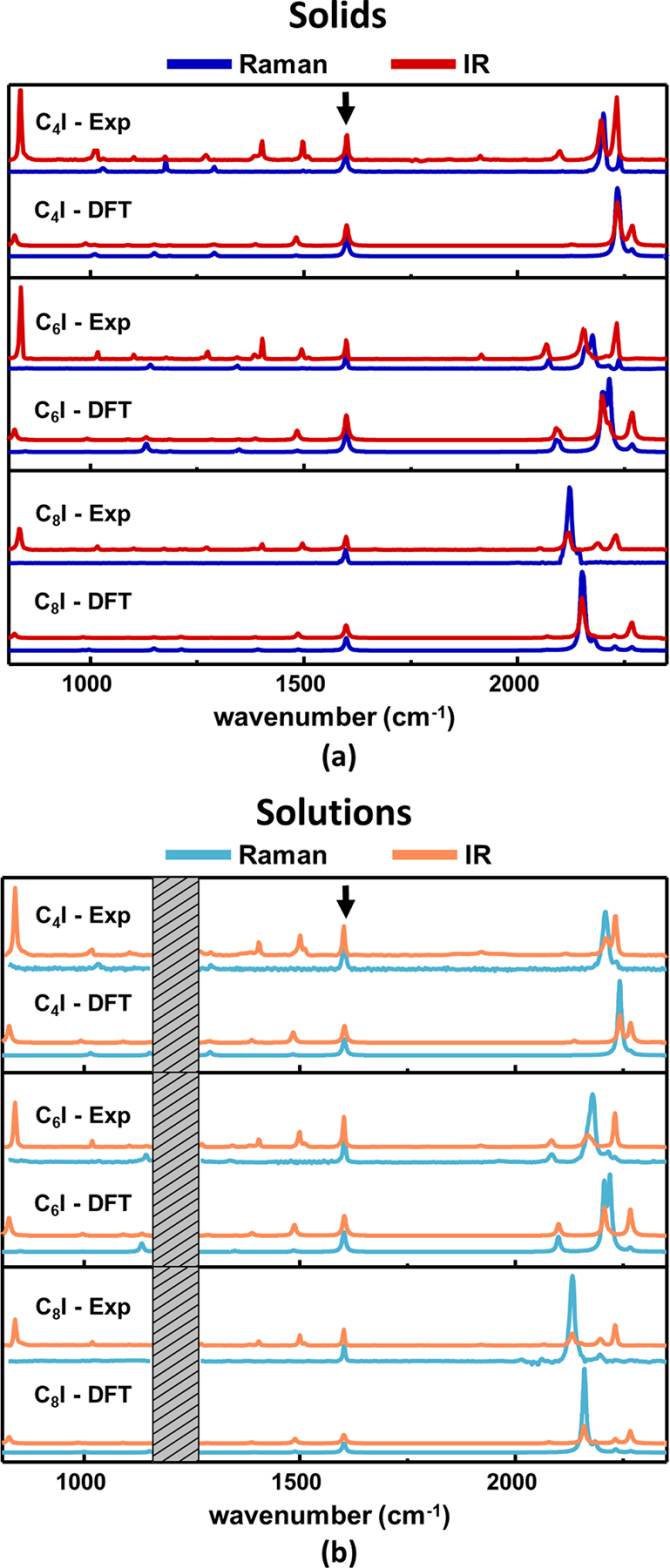
Comparison between the FTIR and FT-Raman spectra of C_*n*_I 1-halopolyynes in solid-state samples (a) and solutions (b). Simulated spectra (DFT) and experimental spectra (Exp) are shown. Experimental powder and solution spectra have been compared with simulations of HT dimers (a) and single molecules (b), respectively. The black arrows indicate the phenyl stretching mode (P-mode) used to normalize the Raman intensities of each spectrum and rescale the DFT computed frequencies (see Experimental Methods). The grayed regions in the solutions panel cover the absorption bands of the solvent (chloroform) which cannot be compensated by the background subtraction.

FTIR and FT-Raman spectra of powders and solutions of iodine-terminated polyynes (C_*n*_I) are shown in Figure 6 as representatives for all the investigated 1-halopolyynes whose spectra are displayed in Figures S2 and S3 in the Supporting Information. Each experimental spectrum is compared to the corresponding DFT simulation, isolated polyynes for experimental spectra of solutions and HT dimers for experimental spectra of solid samples (as discussed in the previous section).

The experimental IR and Raman spectra show intense peaks in the 1800–2300 cm^–1^ spectral region corresponding to polyynes’ Raman-active ECC mode. The simultaneous activation of the ECC mode in both Raman and IR arises from asymmetric polar terminations that polarize the CC bonds of the sp-carbon backbone. In a push–pull system, the charge transfer between the two end groups determines a sizable polarization of the conjugated backbone, leading to similar intensity pattern in Raman and IR spectra, both showing a dominant ECC band.^21^ In our systems, Raman spectra show ECC mode largely overcoming other peaks’ intensity while its IR intensity is comparable to several other vibrational bands, thus excluding a push–pull behavior in agreement with the analysis of IR charges.

The experimental Raman spectra reported in Figure 6 were normalized against the phenyl stretching mode (P-mode) at about 1600 cm^–1^, used as an internal reference, as in our previous work.^21^ Indeed, it experiences just a smooth increase of its Raman intensity with the sp-carbon chain length (see Figure S4a in the Supporting Information) even if it couples with the sp-carbon chain and is affected by its π-electron conjugation. The Raman intensity of the ECC mode shows rapid growth with an increasing chain length. This trend nicely parallels the DFT computed Raman activities of the ECC mode, illustrated in Figure S5a in the Supporting Information. The choice of the halogen termination slightly affects the Raman cross-section of the ECC band that is systematically higher for larger electron-donor capability of the halogen, namely for C_*n*_I, as shown in Figure S5a in the Supporting Information.

On the contrary, the DFT computed IR intensity of the ECC mode displayed in Figure S5b in the Supporting Information does not show any systematic trend. The computed vibrational eigenvectors demonstrate that the ECC mode is kinetically coupled to the stretching of the C–X bond and, in some cases, to vibrations localized on the C–CN bond. Because of the high polarity of the bonds belonging to the end groups, these couplings affect the IR intensity of the ECC mode in a rather complex way, which cannot be related to the conjugation properties of the sp-carbon chain or the kind of halogen termination.

**Figure 7:**
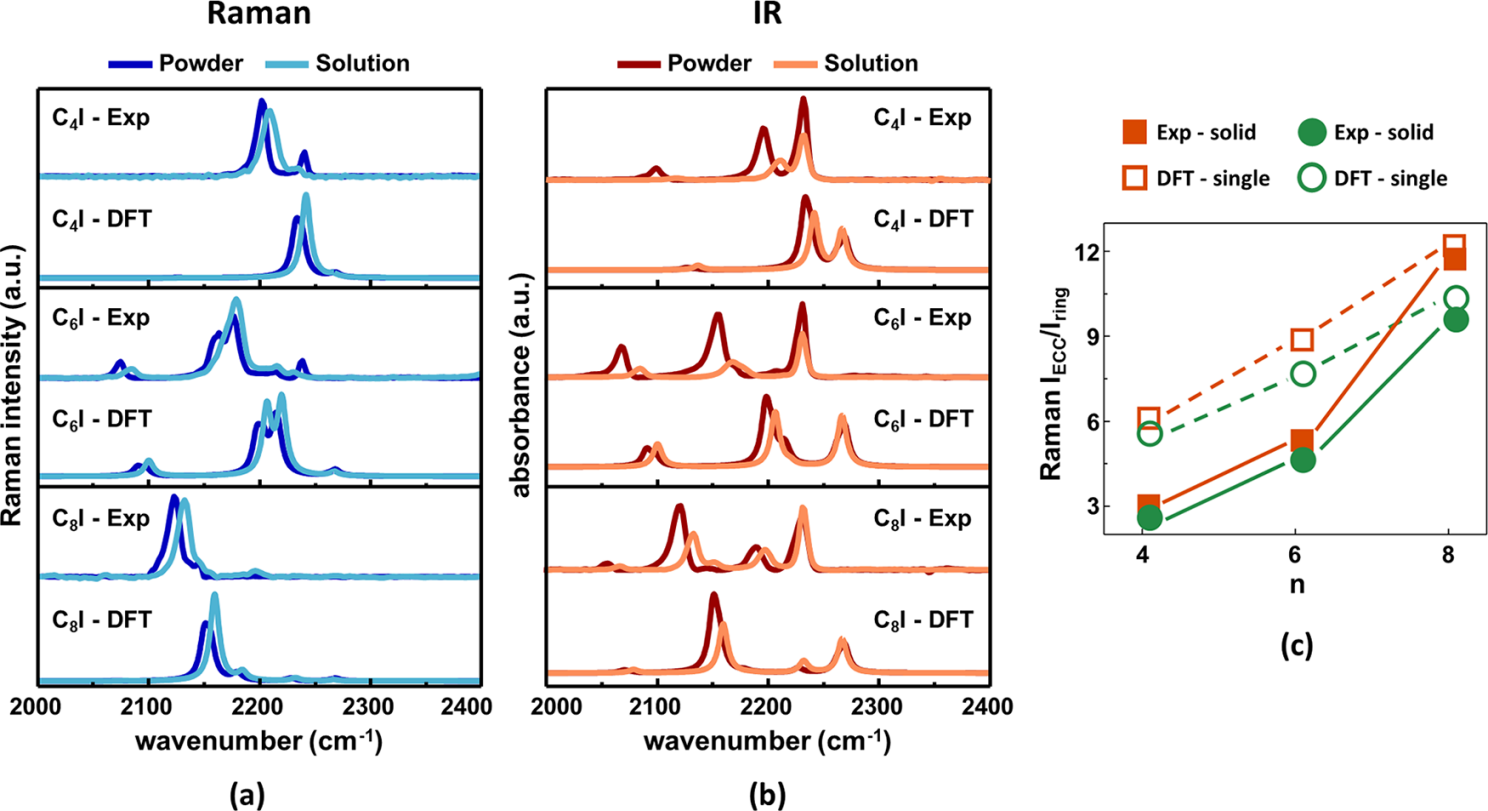
Comparison between the FT-Raman (a) and FTIR (b) spectra of C_*n*_I 1-halopolyynes in the solid state and solutions. Simulated (DFT) and experimental (Exp) spectra are shown. Experimental powders and solution spectra have been compared with those simulated for HT dimers and single molecules, respectively. (c) DFT and experimental intensity ratios between the ECC and the P-mode for the C_*n*_I series as a function of the sp-chain length (*n*). Theoretical values refer to the isolated molecules and their HT dimers, while the experimental data are obtained from integrated band intensities of the spectra of solutions and powders.

Comparing solid and solution spectra and analyzing the results obtained from DFT calculations of isolated molecules and HT dimers (Figure 7), we did not observe any loss or appearance of peaks passing from solutions to powders. However, in some cases, vibrations close in frequency and clearly distinguishable in powders become a single broader band in solution spectra due to the typical band-broadening of solutions in IR spectra.^84^ Moreover, in the experimental spectra of powders, the ECC band and the other neighboring spectral features (above 2000 cm^–1^) exhibit a systematic down-shift of about 12 cm^–1^ compared to solutions. This phenomenon is highlighted in a close-up of the ECC region (2000–2300 cm^–1^) of C_*n*_I molecules, as reported in Figure 7a,b. The observed frequency shifts are due to intermolecular interactions, as predicted by theory. Indeed, the DFT-calculated spectra of HT dimers in Figure 7a,b exhibit a frequency down-shift of about 6 cm^–1^ of the ECC band passing from isolated molecules to dimers, thus suggesting intermolecular interactions occurring in solid-state and not in solution. This interpretation is further confirmed by the spectra of solutions measured at different polyynes concentrations (from 10^–2^ to 10^–6^ M), not showing significant differences (see Figures S6 and S7 in the Supporting Information). Regarding band intensities, the relative IR intensity of the ECC band in solutions is lower than that in powders. This phenomenon is well reproduced by calculations of HT dimers and isolated molecules (see Figure S5b in the Supporting Information). We interpret this decreasing trend as related to the strong intermolecular interactions that affect the electronic structure in the solid state, as suggested by the non-negligible charge transfer between the two molecules composing the dimer. In analogy with hydrogen-bonded complexes, non-negligible charge fluxes can occur during molecular stretching vibrations between the two molecules of the dimer. This phenomenon can explain the increase of the ECC modes’ intensity in the IR spectra of solid-state samples. Furthermore, as in the case of IR intensities, the theoretical Raman activities of the ECC mode grow in HT dimers compared to isolated molecules (see Figure S5a in the Supporting Information). However, this feature is not appreciated experimentally (see Figures 6, S2, and S3 in the Supporting Information) because of the normalization with the P-mode. Indeed, we observe a slight decrease of the *I*_ECC_/ *I*_ring_ ratio in powders compared to solutions, for all polyyne lengths, as shown in Figure 7c. This behavior is observed both experimentally and with calculations. According to DFT calculations, the Raman intensity of the P-mode increases due to the formation of the halogen bond. The calculations for the HT dimer show the existence of two different transitions, close in frequency, associated with phenyl stretching of the donor and acceptor polyynes, respectively. These transitions give rise to a strong band, which is the superposition of the two components, the weaker one having approximately the same Raman activity as that of the isolated molecule. On the contrary, the intensity of the P-mode of the acceptor molecule (the one sharing the CN group) increases because of the significant perturbation of the charge density at the phenyl-CN end involved in the halogen bond.

### FTIR and FT-Raman Analysis of the Vibrational Bands of 1-Halopolyynes

**Figure 8:**
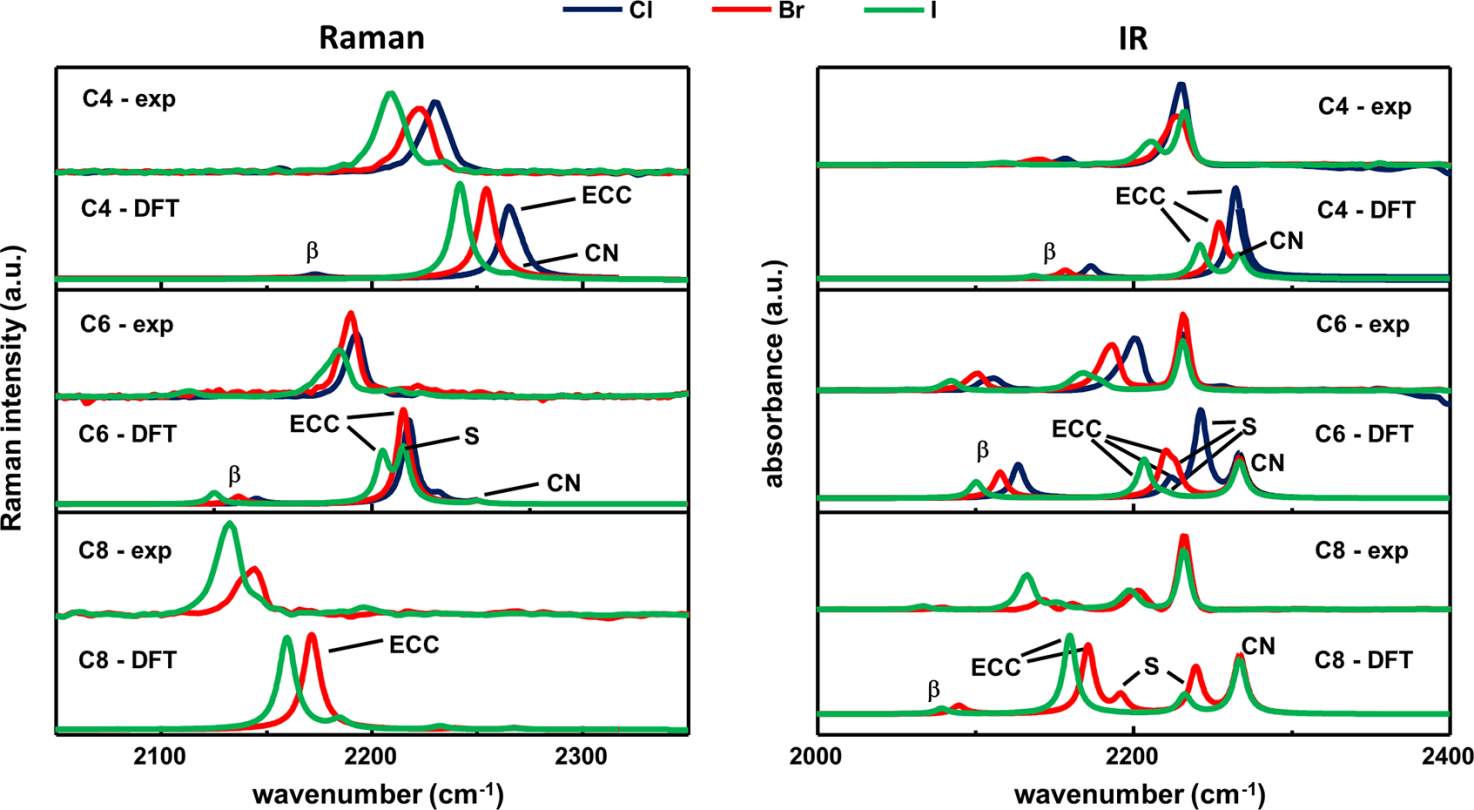
ECC spectral region of the FT-Raman (on the left) and FTIR (on the right) spectra of 1-halopolyynes grouped by their chain length. A comparison between the simulated spectra of the monomers (DFT) and the experimental (Exp) spectra of the solutions is reported. The normal modes discussed in the main text are highlighted by different labels.

In this section, we discuss the main vibrational bands, including some minor features, of 1-halopolyynes, which are listed in Table 3 with their experimental frequencies. We observe nontrivial behavior of some normal modes in the ECC region around 2200 cm^–1^ (see Figure 8) and the functional group region at 900–1700 cm^–1^ (see Figure S8 in the Supporting Information). The frequency range around 2200 cm^–1^ is dominated by the intense ECC mode, whose position shifts at lower frequencies by changing the halogen termination (from Cl to I) and the chain lengths (from 4 to 8 sp-carbon atoms).

**Table 3:** Frequencies (cm^–1^) of the Main Vibrational Modes Observed in FT-Raman and FTIR Spectra of 1-Halopolyynes

	C_4_			C_6_			C_8_	
mode	Cl	Br	I	Cl	Br	I	Br	I
CN			2232^R^^,^^I^	2233^R^^,^^I^	2230^R^^,^^I^	2232^R^^,^^I^	2232^I^	2232^I^
S″							2203^I^	2197^I^
S′				2200^I^	2191^I^	2179^I^	2161^I^	2152^I^
ECC	2231^R^^,^^I^	2222^R^^,^^I^	2208^R^^,^^I^	2191^R^^,^^I^	2186^R^^,^^I^	2174^R^^,^^I^	2142^R^^,^^I^	2132^R^^,^^I^
β	2156^I^	2140^I^	2117^I^	2111^I^	2101^I^	2084^I^	2079^I^	2067^I^
P	1604^R^^,^^I^	1602^R^^,^^I^	1602^R^^,^^I^	1602^R^^,^^I^	1604^R^^,^^I^	1603^R^^,^^I^	1603^R^^,^^I^	1602^R^^,^^I^
M	1308^R^^,^^I^	1297^R^^,^^I^	1291^R^^,^^I^	1362^R^	1354^R^	1350^R^	1399^R^	1395^R^
Z	1070^R^	1043^R^	1032^R^	1147^R^	1140^R^	1133^R^	1222^R^	1216^R^

R Observed in FT-Raman spectra.

I Observed in FTIR spectra.

The peak at approximately 2230 cm^–1^ for all 1-halopolyynes (labeled with CN in Figure 8) is assigned to the stretching of the cyano group of the cyanophenyl. Indeed, since the CN group is far from X and is “shielded” by the phenyl ring, it is only slightly affected by the change in halogen termination and by sp-chain length. The CN and ECC peaks appear as well separated doublet in C_4_I, C_6_X, and C_8_X series, while they overlap in C_4_Cl and C_4_Br.

Even if symmetry selection rules relax due to the asymmetric end groups, several normal modes can be detected only in the IR spectra because of their weak Raman activity. Localized vibrations of polar groups, such as CN stretching, give larger IR absorptions, while the most intense Raman bands are usually due to collective vibrations of nonpolar or moderately polar groups.

The β-mode highlighted in Figure 8 is associated with an out-of-phase C≡C stretching vibration.^85^ Similar to the ECC band, the β-band, clearly visible in the IR spectra, downshifts as a function of chain length and with the halogen termination. Longer chains show Raman and IR transitions associated with another normal mode (S-mode) consisting of longitudinal sp-carbon chain vibrations. The number of observable S modes is *m* = *N*-2, where *N* is the number of triple bonds in the chains.

In the region below 1600 cm^–1^, the IR spectra of 1-halopolyynes show many marker bands, ascribed to modes involving CC stretching of the quasi-single bonds of the sp-carbon chain and end groups vibrations. Two peaks at around 1400 and 1500 cm^–1^ (see Figures 6 and S9 in the Supporting Information) are related to different CC and CH bending vibrations of the phenyl group. They are not affected by the halogen termination or the number of sp-carbon atoms. Both these peaks show a satellite feature not predicted by calculations of isolated molecules or HT dimers. These satellite bands are not related to solid-state effects (e.g., crystal splitting) since they are present even in low-concentration solutions (see Figure S9 in the Supporting Information). These components could be explained as combination bands involving two normal modes at lower frequencies, as suggested in Figure S10 and Table S3 in the Supporting Information.

The intense peak observed in the IR spectra of all 1-halopolyynes at 840 cm^–1^ (see Figure 6) is not Raman-active because of the symmetry selection rules in the presence of a symmetry plane. It is related to the out-of-plane vibration of the CH bonds of the phenyl ring of the cyanophenyl group. This peak shows an impressive intensity in the experimental spectra, occasionally overcoming the ECC peak (see C_4_I and C_6_I in Figure 6), while DFT spectra (Figure 6) predict a weaker intensity. This is due to the exceedingly high IR intensity calculated for the P band used for normalization, as deduced according to the following observations. We have already noticed that both ECC modes and, to a lesser extent, the P-mode increase their DFT Raman activities with increasing conjugation length (see Figures S4 and S5 in the Supporting Information). Because of the presence of polar terminations, both the ECC and the phenyl ring stretching modes gain IR intensity (see Figure S4a,b in the Supporting Information). The mechanism of IR activation is the same as that described in the case of push–pull polyynes and polyenes.^21^ Moreover, it is well known that DFT simulations overestimate both the extent of π-electron delocalization and the Raman intensities of collective vibrations of conjugated CC bonds, such as the ECC mode.^68,85^ The above arguments suggest that the DFT-calculated Raman and IR intensities of the ECC and P modes are overestimated. For this reason, the calculated IR spectrum, normalized on the P-mode, features a highly reduced IR intensity of the out-of-plane mode (at 840 cm^–1^) compared to the experimental spectrum (see Figure 6).

**Figure 9:**
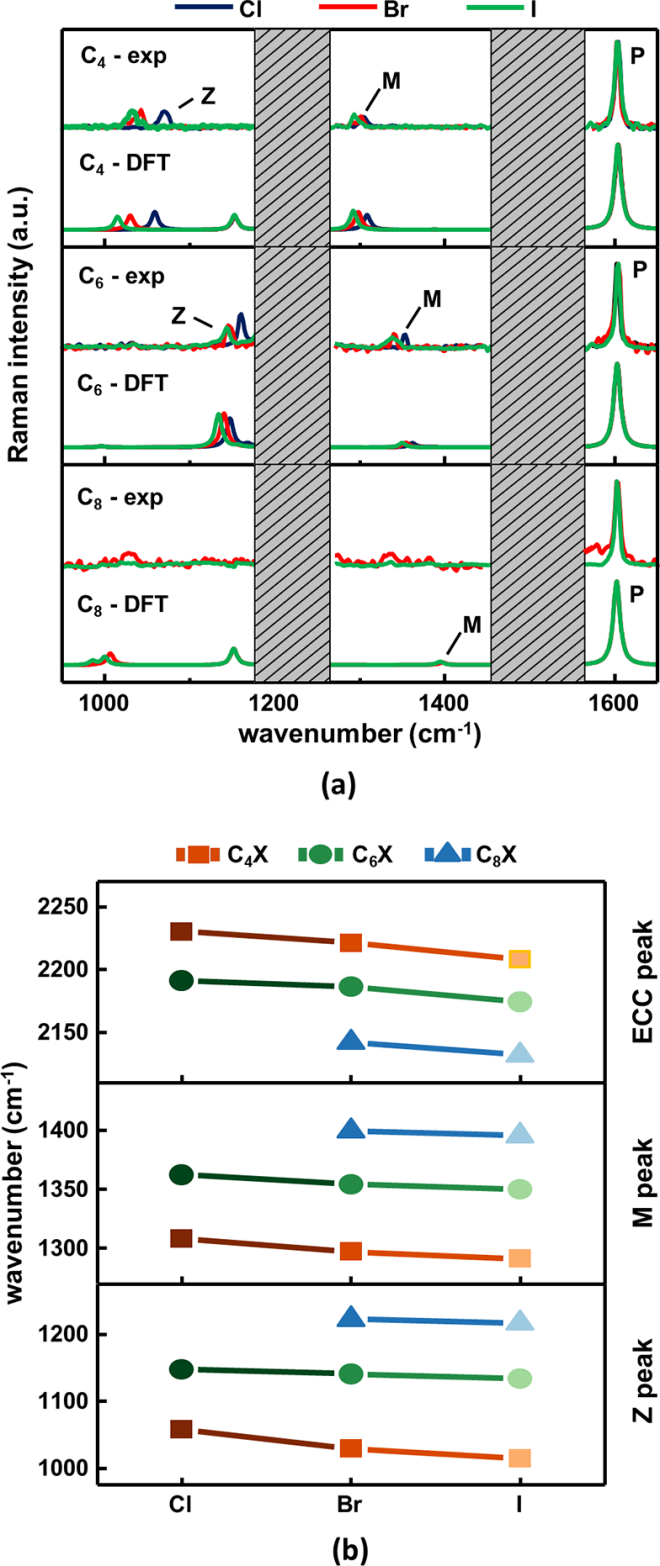
(a) Low-frequency region (900–1700 cm^–1^) of the FT-Raman spectra of 1-halopolyynes grouped by their chain lengths. A comparison between the simulated (DFT, single molecules) and experimental (Exp) spectra (solutions) is shown. The normal modes discussed in the main text are highlighted by different labels. (b) Frequencies of the ECC (exp), M (DFT), and Z (DFT) modes of 1-halopolyynes, extracted from Raman spectra.

In addition to the P-mode, we noticed two other Raman-active normal modes in the 900–1700 cm^–1^ spectral region. They involve vibrations of the sp-carbon chains coupled to in-plane bending vibrations of the phenyl ring. They are very sensitive to BLA variations and hence to the π-electron conjugation of the systems, as highlighted in Figure 9a. We name the higher frequency band, whose position varies between 1300 and 1400 cm^–1^, the M peak. It is mainly associated with the stretching of single bonds of the sp-carbon chain (see Figure S11 in the Supporting Information). The M-mode, compared to the P-mode, weakens from C_4_X to C_8_X. The lower frequency band, named Z peak, in the 1000–1200 cm^–1^ spectral region, is characterized by single CC bond stretchings coupled to the breathing vibration of the phenyl ring (reported in Figure S11 in the Supporting Information).

Figure 9a shows that the frequency of the M and Z peaks downshifts by increasing the electron-donor capability of the halogen termination (from Cl to I), while upshifts for longer chains. The trend of variation of the M and Z peaks with chain lengths is opposite of that of the ECC and β modes. This behavior is due to the different contributions by single and triple CC bond stretching in these modes. ECC and β modes involve stretching of both single and triple bonds, whereas the M and Z modes feature oscillations localized on single bonds. By increasing the chain length, the BLA decreases, so the single bonds become shorter and stronger, with a higher diagonal C–C stretching force constant. This results in an increase of the M and Z mode frequencies with the chain length. Instead, the triple bonds elongate while increasing the conjugation length (and decreasing BLA), and their diagonal stretching force constant decreases, thus explaining the lowering of the stretching frequency of the ECC and β modes. For a more accurate description, other effects such as nondiagonal interactions between CC bonds at increasing distances along the conjugated chain, the kinetic coupling with end groups, and the effects of different effective masses of the oscillator should be taken into account. However, the essence of the frequency behavior is already well rationalized considering only the modulation with BLA of the diagonal CC stretching force constants.

In conclusion, the position of the ECC, M, and Z modes reported in Figure 9b shows the existence of a relationship between frequencies and halogen terminations and/or chain lengths. Also in this case, the impact of the halogen is weaker than that of the chain length. In particular, considering the C_*n*_Br polyynes as a reference, we measured an average frequency variation of −0.3% for C_*n*_Cl and 0.5% for C_*n*_I (ECC peak), −0.7% for C_*n*_Cl and 0.3% for I (M peak), and −1.5% for C_*n*_Cl and 1% for C_*n*_I (Z peak). On the contrary, considering C_6_X as a reference, we estimated an average frequency variation with the chain length of −1.6% for C_4_X and 2.0% for C_8_X (ECC peak), −4.2% for C_4_X and 3.3% for C_8_X (M peak), and −9.5% for C_4_X and 7.5% for C_8_X (Z peak).

## Conclusions

We investigated the optical and vibrational properties of 1-halopolyynes (i.e., C_*n*_X) with different halogen terminations (Cl, Br, and I) and chain lengths (4, 6, and 8 sp-carbon atoms), together with their crystal structures. The asymmetric and polar end groups are responsible for the permanent dipole moment and the polarization of the CC bonds of the sp-chain of these systems. This allowed us to detect the IR and Raman signals of the most characteristic vibrations, such as the ECC and β modes and other marker bands. The vibrational spectra of solid-state samples and diluted solutions show a non-negligible shift of the ECC mode. We modeled this shift using head-to-tail (HT) dimers, characterized by a strong halogen bond between the CN and halogen terminations. By diluting the powders in a proper solvent (chloroform), the dimer-based crystal structure is lost, and the vibrational spectra can be explained using theoretical simulations for isolated molecules. UV–vis absorption spectra, provide a further characterization of these systems in solution and allow us to identify the role of different halogen terminations and chain lengths on the π-electron conjugation along the molecular backbone. Beyond the well established relationship between polyynes conjugation and chain length, we demonstrate that an increase in the electron-donating ability of the halogen termination (from Cl to I) slightly reduces the energy gap of these systems. These phenomena observed by experimental UV–vis absorption spectra and predicted by DFT simulations affect also the FTIR and FT-Raman spectra modulating the ECC frequency. Furthermore, analyzing the behavior of the ECC mode and two other vibrations (M and Z modes), we confirmed that the chain length variation, through the tuning of the conjugation length, rather than the halogen termination, is more effective in the modulation of the spectroscopic response.

DFT simulations carried out both on isolated molecules and on dimers allow us to understand the behavior of 1-halopolyynes with different chemical structures. The positive atomic charges of halogen atoms, derived from DFT computed APTs, justify the fact that the halogen terminations can lead to specific and directional intermolecular interactions thanks to the possibility of forming halogen bonding between adjacent halogenated sp-carbon chains. The actual formation of halogen bonding is discussed in the light of the crystal structures of 1-halopolyyne, based on XRD single-crystal determination previously published for the C_*n*_I series, and on the recent XRD determination for C_4_Cl, C_4_Br, C_6_Cl, and C_6_Br, reported here for the first time. Halogen bonding is observed for C_4_Cl, C_4_Br, and for the whole series C_*n*_I which form linear head-to-tail dimers in the crystal. Instead, C_6_Br and C_6_Cl show a completely different herringbone packing motif, which can be explained by the competition between the formation of a rather weak halogen bond and effective van der Waals interactions between adjacent π-electron systems. The solid-state structure characterized by halogen bonding affects several properties of 1-halopolyynes (e.g., a reduction of the energy gap), thus suggesting that solid-state intermolecular interactions can play a non-negligible role in the tunability of the physics of conjugated systems. The above conclusion can be generalized to more complex halogenated and π-conjugated molecules, thus paving the way for future development and understanding of halogen-terminated carbon wires.

## Data Availability

Deposition numbers CCDC2281261–2281264 contain the supplementary crystallographic data for this paper. These data are provided free of charge by the joint Cambridge Crystallographic Data Centre and Fachinformationszentrum Karlsruhe Access Structures service.

## Supporting Information Available

UV–vis, FTIR, and FT-Raman spectra for all the halogenated polyynes; dipole moments, interaction energies, electronic charges, and normal modes derived from DFT for all the halogenated polyynes; synthesis of C_6_Cl and C_8_Br with ^1^H and ^13^C NMR spectra; and Hirshfeld surfaces for C_*n*_I, C_4_Cl, C_6_Cl, C_4_Br, C_6_Br (PDF)
XRD crystallographic data for C_4_Cl, C_4_Br, C_6_Cl, and C_6_Br (CIF)

## Supplementary Material

jp3c07915_si_001.pdf

jp3c07915_si_002.cif
