# Supplementary material for: Impact of Halogen Termination and Chain Length on π-Electron Conjugation and Vibrational Properties of Halogen-Terminated Polyynes
Source: J Phys Chem A. 2024 Mar 20;128(14):2703–16. doi: 10.1021/acs.jpca.3c07915 (PMC11017249; doi:10.1021/acs.jpca.3c07915)
Supplement: Supplementary file 1 — jp3c07915_si_001.pdf [file jp3c07915_si_001.pdf]

<u>Supporting Information:</u>

Impact Of Halogen Termination And Chain Length On π-Electron Conjugation And Vibrational Properties Of Halogen Terminated Polyynes

*Simone Melesi [a], Pietro Marabotti [a,d], Alberto Milani [a], Bartłomiej Pigulski [b], Nurbey Gulia [b], Piotr Pińkowski [b], Sławomir Szafert [b], Mirella Del Zoppo [c], Chiara Castiglioni [c], Carlo S. Casari [a]**

[a] Department of Energy, Micro and Nanostructured Materials Laboratory - NanoLab, Energy, Politecnico di Milano, Via Ponzio 34/3, Milano 20133, Italy

[b] Faculty of Chemistry, University of Wrocław, 14 F. Joliot-Curie, Wrocław 50-383, Poland

[c] Department of Chemistry, Materials and Chemical Engineering "Giulio Natta", Politecnico di Milano Piazza Leonardo da Vinci 32, Milano 20133, Italy

[d] Institut für Physik and IRIS Adlershof, Humboldt Universität zu Berlin, 12489 Berlin, Germany

Corresponding author email: *carlo.casari@polimi.it*

Table of contents

i. UV-Vis, FT-IR, FT-Raman, and DFT data 2

ii. Synthesis and characterization of new polyynes 13

iii. X-ray crystallography details 16

iv. *I*nteraction energies and Hirshfeld surface analysis 23

v. References 28

i. UV-Vis, FT-IR, FT-Raman, and DFT data

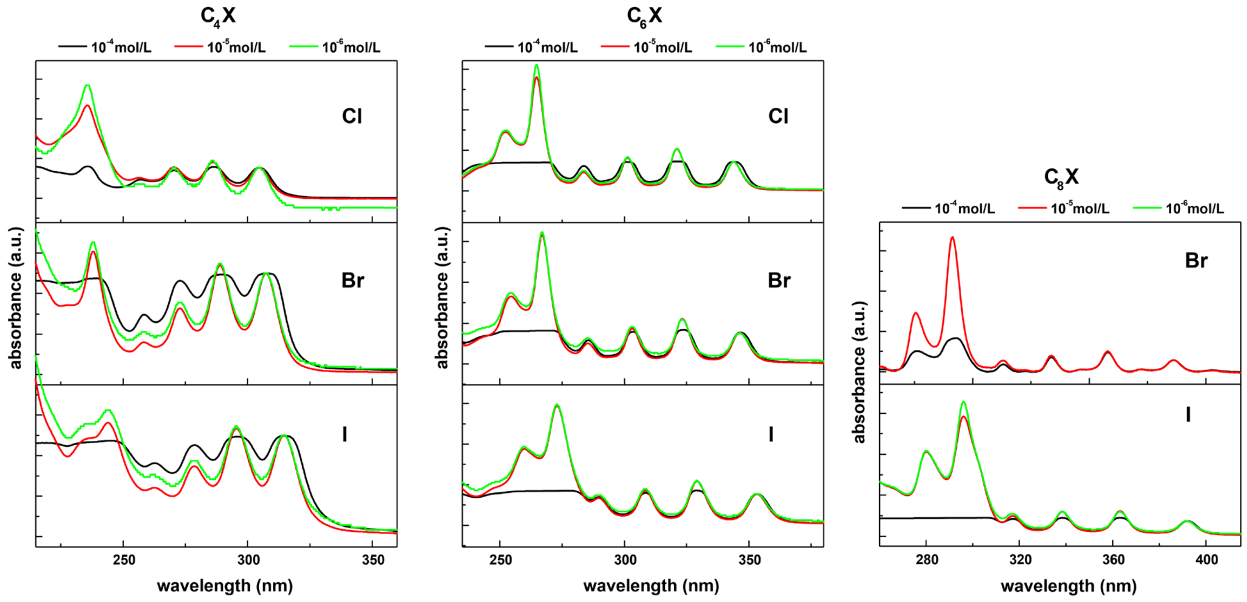

Figure S1 UV-Vis absorption spectra of 1-halopolyynes at different concentrations (from 10^{-4} mol/L to 10^{-6} mol/L). Molecules are grouped by chain length and halogen termination and indicated with C_nX, where n is the number of carbon atoms in the chain (n= 4, 6, 8) and X is the halogen (X= Cl, Br, I) termination. Spectra at 10^{-4}mol/L are too concentrated, and saturation of the signals is visible in almost all spectra (except for C4Cl).

Table S1 (a) DFT-calculated dipole moments (in Debye) of C_nX 1-halopolyynes and (b) atomic IR charges (in electrons) on the halogen atom (X =Cl, Br, I), carbon atom (C1) bonded to the halogen termination (X), and nitrogen atom (N). These charges are obtained from Atomic Polar Tensors calculated by DFT simulations carried out for isolated molecules. (c) Atomic IR charges (in electrons) of N and halogen atoms for isolated molecules and the two (donor and acceptor) molecules of the head-to-tail (HT) dimer of C_4X (X= Cl, Br) and C_6I and C_8I species. For the same species, the group charge of the sp chain and the total charge of the two partner molecules of the dimer are reported. Δq values are computed considering the isolated molecule as a reference.

(a)

Dipole moment (Debye)			
**n**	**Cl**	**Br**	**I**
**4**	4.185	4.332	4.718
**6**	4.233	4.395	4.824
**8**	4.278	4.453	4.919

(b)

	q_X (e)			q_C1 (e)			q_N (e)		
**n**	**C_nCl**	**C_nBr**	**C_nI**	**C_nCl**	**C_nBr**	**C_nI**	**C_nCl**	**C_nBr**	**C_nI**
**4**	0.139	0.148	0.193	-0.171	-0.146	-0.230	-0.303	-0.303	-0.303
**6**	0.152	0.158	0.202	-0.190	-0.150	-0.222	-0.301	-0.301	-0.301
**8**	0.152	0.161	0.208	-0,208	-0.142	-0.219	-0.300	-0.300	-0.300

(c)

**C_4Cl - isolated**		**C_4Cl – HT - acceptor**			**C_4Cl - HT -donor**		
	**q(e)**		**q(e)**	**Δq(e)**		**q(e)**	**Δq(e)**
**N**	-0.303	**N**	-0.282	0.021	**N**	-0.306	-0.003
**Cl**	0.139	**Cl**	0.141	0.002	**Cl**	0.140	0.001
**sp-chain**	-0.206	**sp-chain**	-0.204	0.002	**sp-chain**	-0.227	-0.021
		**Acceptor molecule**	0.034		**Donor molecule**	-0.034	
**C_4Br - isolated**		**C_4Br – HT - acceptor**			**C_4Br - HT - donor**		
	**q(e)**		**q(e)**	**Δq(e)**		**q(e)**	**Δq(e)**
**N**	-0.303	**N**	-0.282	0.021	**N**	-0.306	-0.003
**Br**	0.148	**Br**	0.151	0.003	**Br**	0.116	-0.032
**sp-chain**	-0.217	**sp-chain**	-0.214	0.003	**sp-chain**	-0.221	-0.004
		**Acceptor molecule**	0.052		**Donor molecule**	-0.052	
**C_6I - isolated**		**C_6I – HT - acceptor**			**C_6I - HT - donor**		
	**q(e)**		**q(e)**	**Δq(e)**		**q(e)**	**Δq(e)**
**N**	-0.301	**N**	-0.252	0.049	**N**	-0.304	
**I**	0.203	**I**	0.208		**I**	0.148	-0.055
**sp-chain**	-0.275	**sp-chain**	-0.269		**sp-chain**	-0.294	-0.018
		**Acceptor molecule**	0.088		**Donor molecule**	-0.088	
**C_8I - isolated**		**C_8I – HT - acceptor**			**C_8I - HT - donor**		
	**q(e)**		**q(e)**	**Δq(e)**		**q(e)**	**Δq(e)**
**N**	-0.300	**N**	-0.252	0.048	**N**	-0.303	
**I**	0.208	**I**	0.213		**I**	0.154	-0.054
**sp-chain**	-0.285	**sp-chain**	-0.277		**sp-chain**	-0.308	-0.023
		**Acceptor molecule**	0.088		**Donor molecule**	-0.088	

Table S2 Calculated DFT values for interaction energies (kcal/mol) of 1-halopolyynes in the head-to-tail (HT) configuration and(in the antiparallel configuration (AP) for the molecules showing halogen bonding.

	**HT dimer<br>Interaction energies (kcal/mol)**	**AP dimer<br>Interaction energies (kcal/mol)**
**C_4Cl**	2.525	1.724
**C_4Br**	3.571	1.888
**C_4I**	5.285	2.230
**C_6I**	5.439	2.834
**C_8I**	5.537	2.964

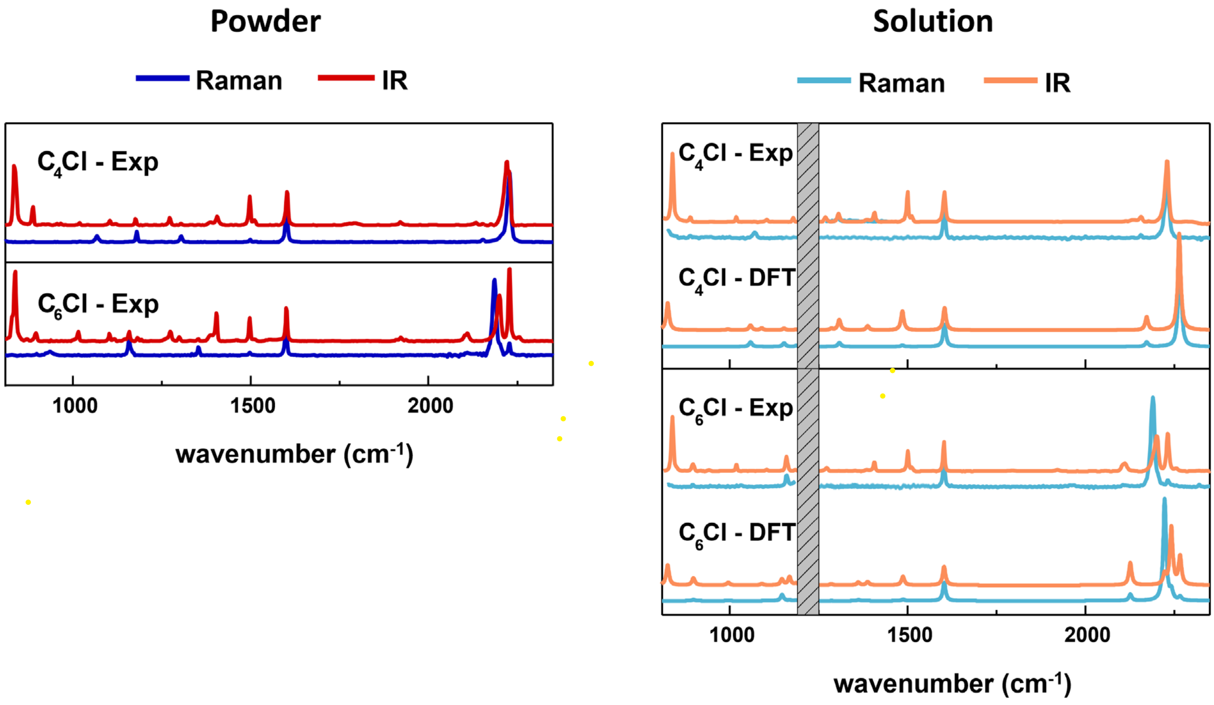

Figure S2 Comparison between the experimental FTIR and FT-Raman spectra of C_nCl 1-halopolyynes in solid-state samples (on the left) and solutions (on the right). Simulated spectra (DFT) for the isolated molecules are compared with the experimental spectra (Exp) of the solutions (on the right). The greyed regions in the right panel cover the absorption bands of the solvent that cannot be compensated by the background subtraction.

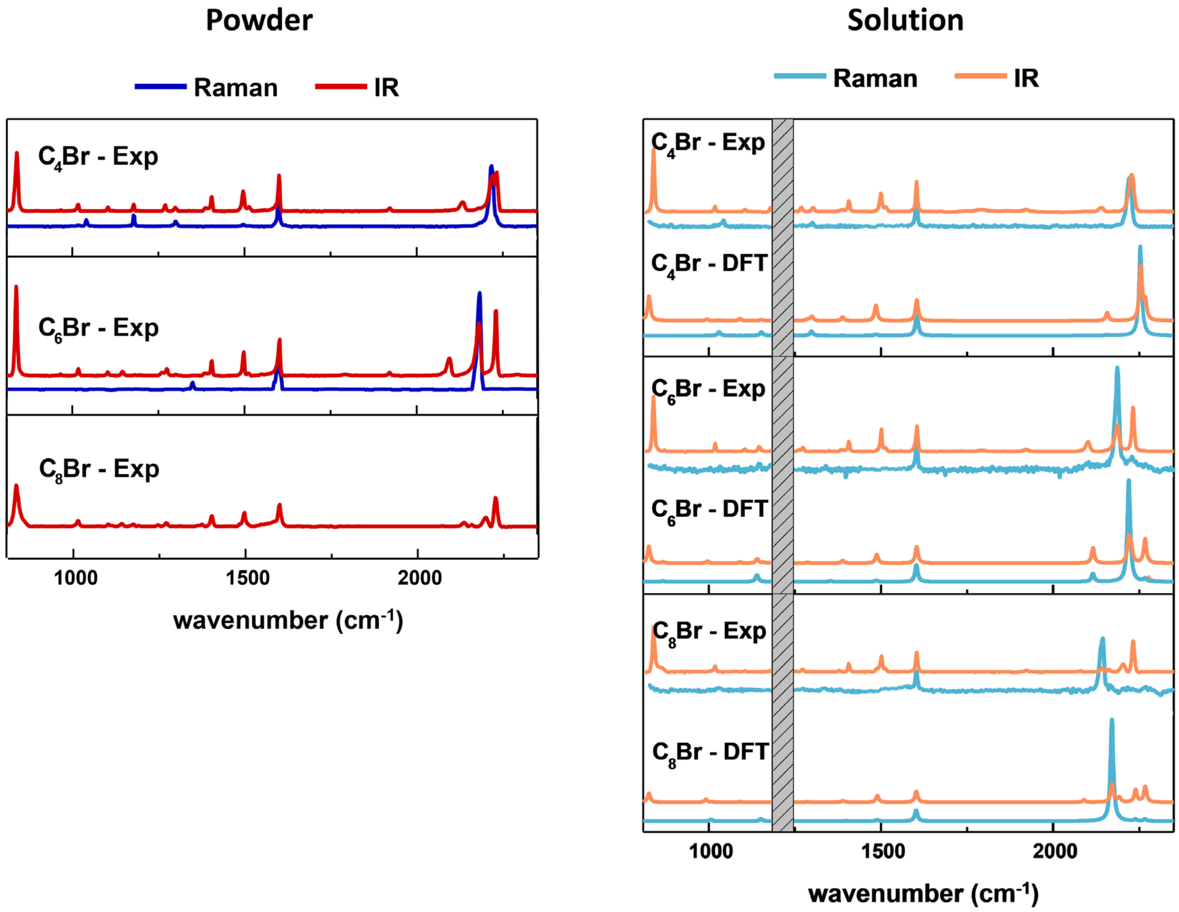

Figure S3 Comparison between the experimental FTIR and FT-Raman spectra of C_nBr 1-halopolyynes in solid-state samples (on the left) and solutions (on the right). Simulated spectra (DFT) for the isolated molecules are compared with the experimental spectra of the solutions (Exp) (right panel). The greyed regions in the right panel cover the absorption bands of the solvent that cannot be compensated by the background subtraction. Solid-state Raman spectrum of C_8Br was not collected due to the very high instability of this molecule.

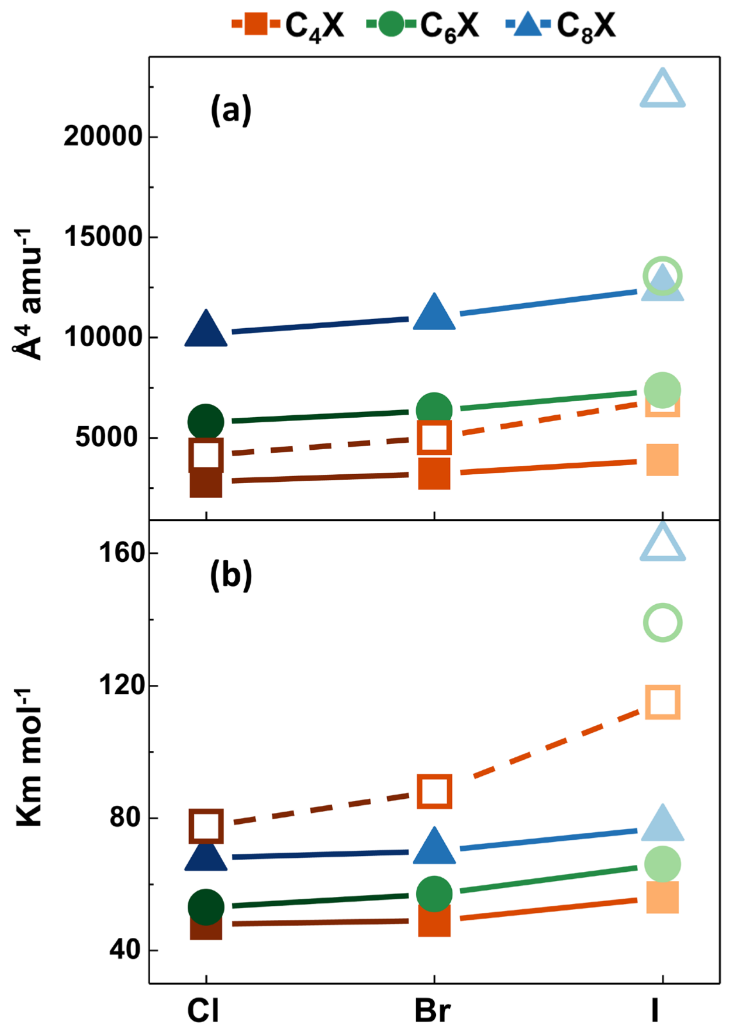

Figure S4 (a) DFT computed Raman (Å^{4}amu^{-1}) and (b) infrared (Km mol^{-1}) activity of the P band for the CnX series. Isolated molecules are represented with solid lines and full symbols while the corresponding HT dimers are represented with dashed lines and empty symbols.

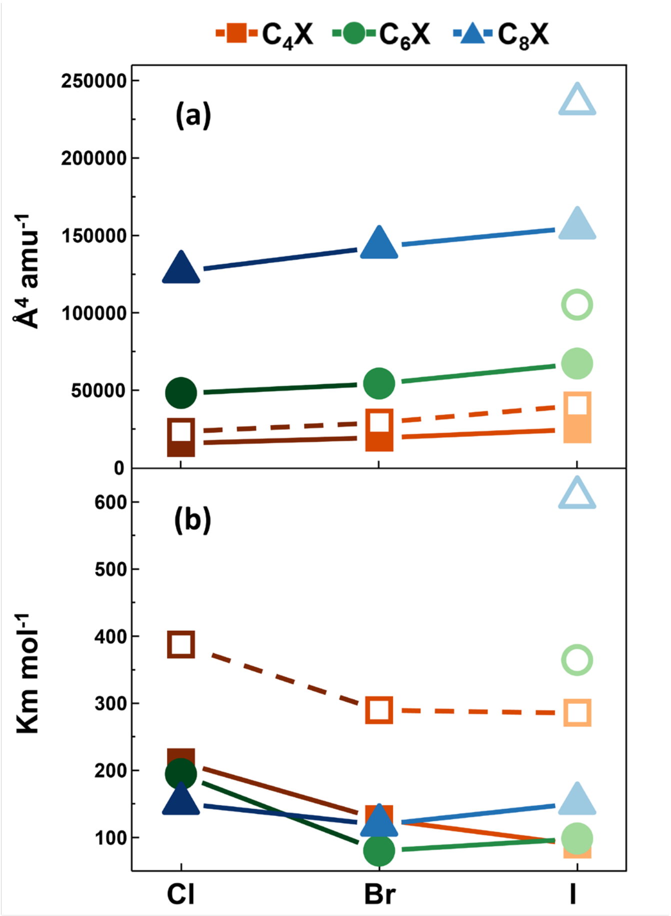

Figure S5 (a) DFT computed Raman (Å⁴amu⁻¹) and (b) infrared (Km mol⁻¹) activity of the ECC band of 1-halopolyynes' (C_nX). Isolated molecules are represented with solid lines and full symbols, while the corresponding HT dimers are represented with dashed lines and empty symbols.

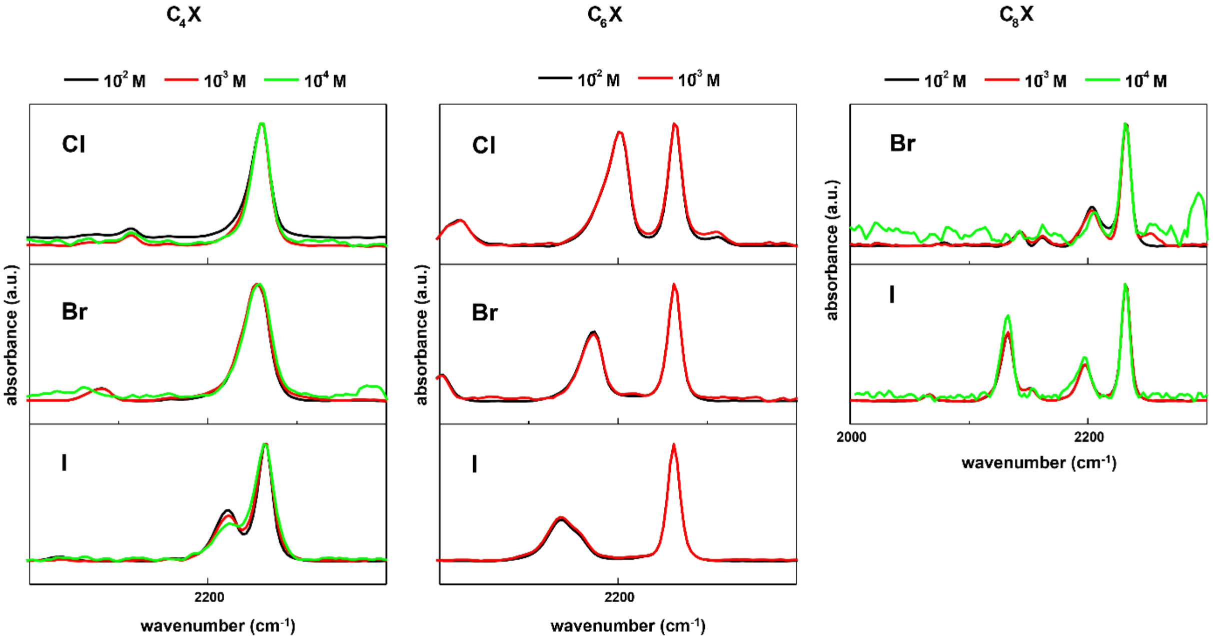

Figure S6 FT-IR spectra of 1-halopolyynes at different concentrations (from 10^{-2} mol/L to 10^{-4}mol/L). Molecules (C$_n$X) are grouped by length and halogen termination.

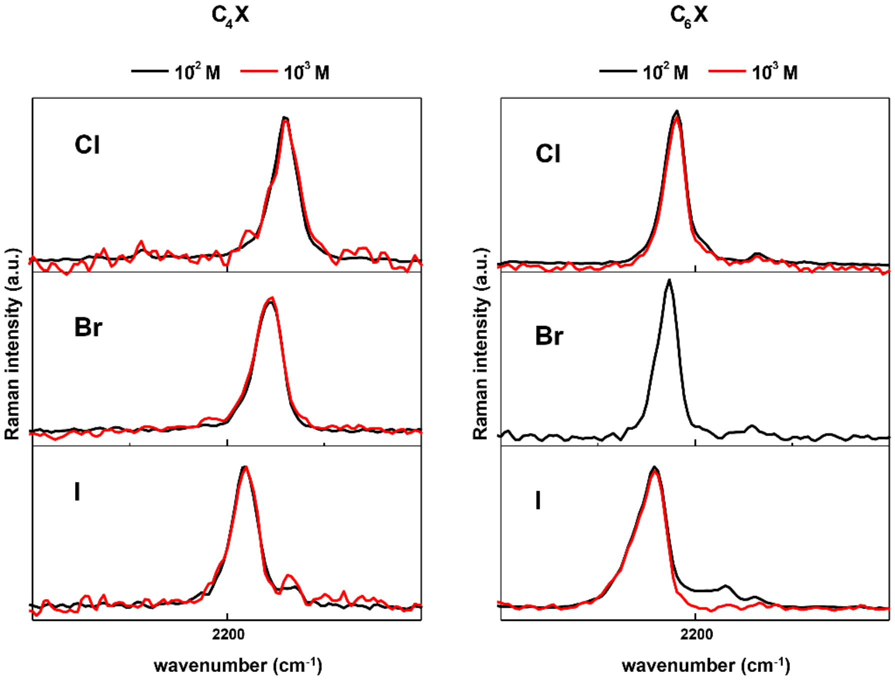

Figure S7 FT-Raman spectra of 1-halopolyynes at different concentrations (10^{-2} mol/L and 10^{-3}mol/L). Molecules (C$_n$X) are grouped by length and halogen terminations. Spectra of C$_6$Br and C$_8$X at low concentrations cannot be recorded due to the instrumental low detection limit and the high instabilities of these molecules.

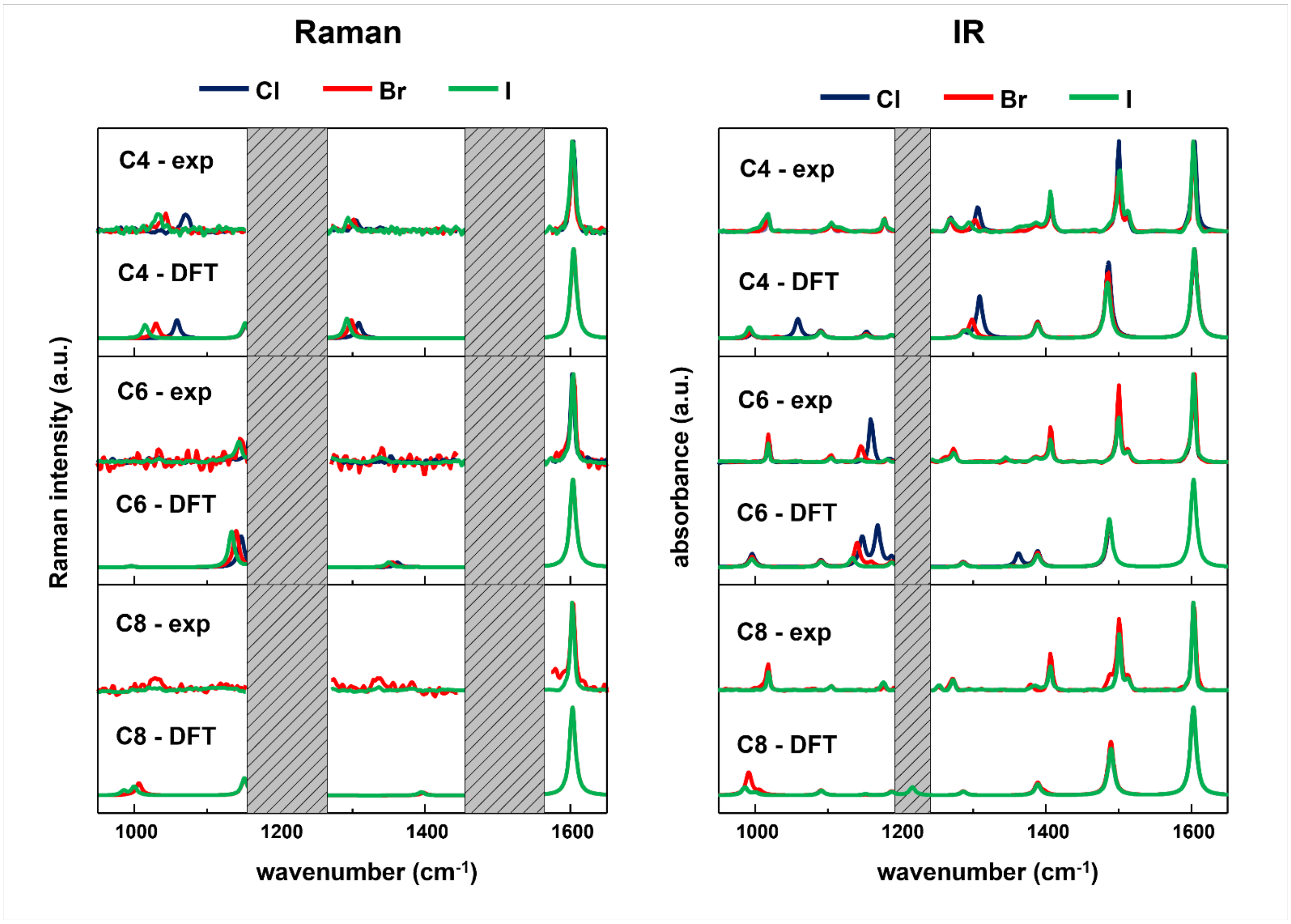

Figure S8 Low-frequency region (900-1700 cm^{-1}) of the FT-Raman spectra (on the left) and FT-IR spectra (on the right) of 1-halopolyynes grouped by their chain lengths. A comparison between the simulated (DFT) spectra of isolated molecules and experimental (Exp) solution spectra is shown.

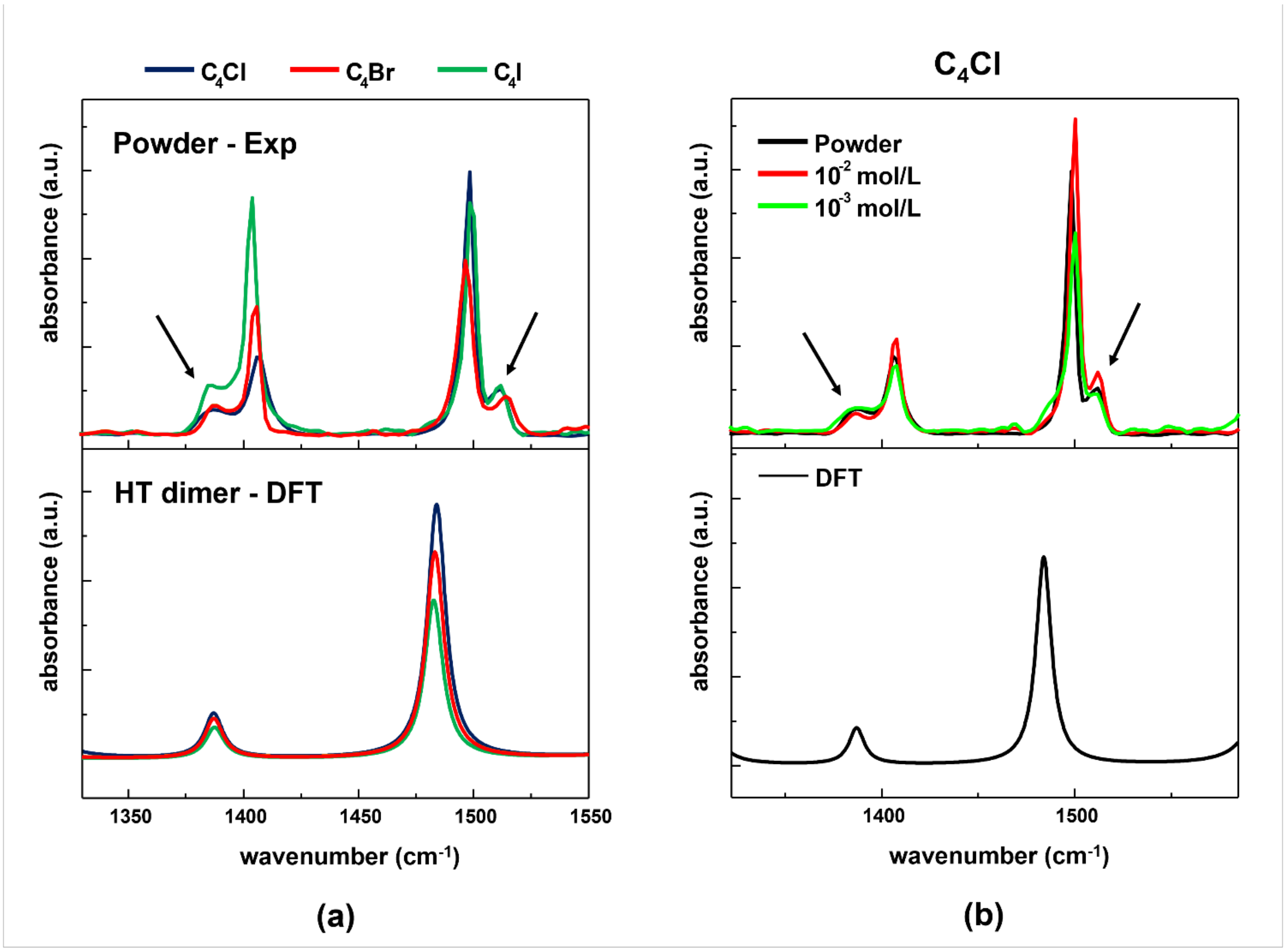

Figure S9 Highlight of the doublets present in the FT-IR spectra of 1-halopolyynes at approximately 1400 and 1500 cm^{-1}. On the left, spectra of solid-state C_4X molecules (where X= Cl, Br, I) are compared with the corresponding calculated DFT spectra of HT dimers. On the right, the C_4Cl molecule has been taken as an example to observe the presence of these doublets even in solution's spectra with different concentrations. The calculated DFT spectrum of isolated C_4Cl is reported for comparison.

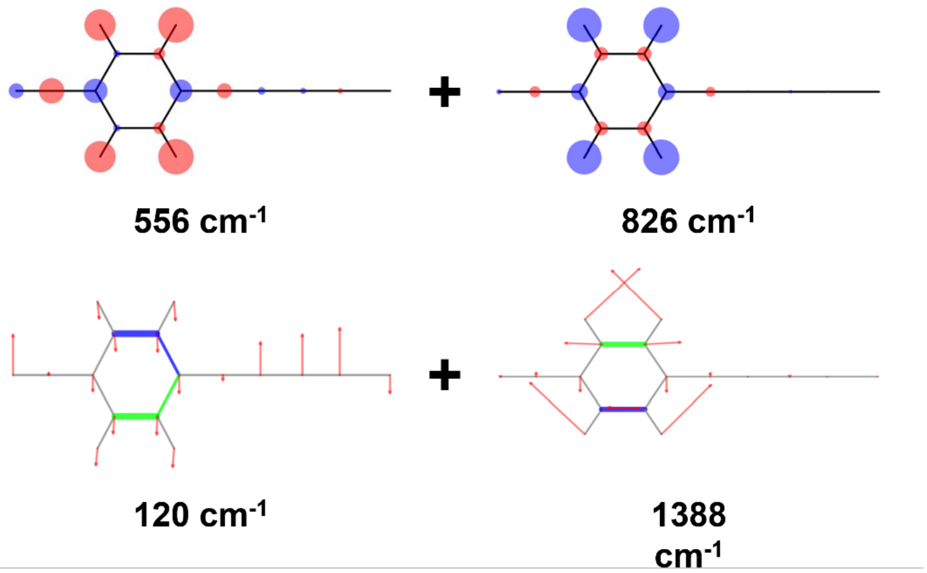

Figure S10 Two possible combinations of normal modes that can explain the presence of the two doublets shown in Fig. S.9. The representations of the vibrational eigenvectors and their frequencies rescaled with the correction factor (see Methods) are reported.

Table S3 Rescaled frequencies of the normal modes that, by combining, can give rise to the two doublets shown in Fig. S.9. The sum of the frequencies is reported. The calculated x, y, and z components of ∂M/∂Q for these normal modes are reported.

**Normal mode #1**				**Normal mode #2**				**Combination**
**Scaled Frequency**	$\partial M/\partial Q$			**Scaled Frequency**	$\partial M/\partial Q$			**Sum of Frequencies**
	**x**	**y**	**z**		**x**	**y**	**z**	
556	0	0	0.73	826	0	0	1.18	1382
120	0	0.36	0	1388	0	0.46	0	1508

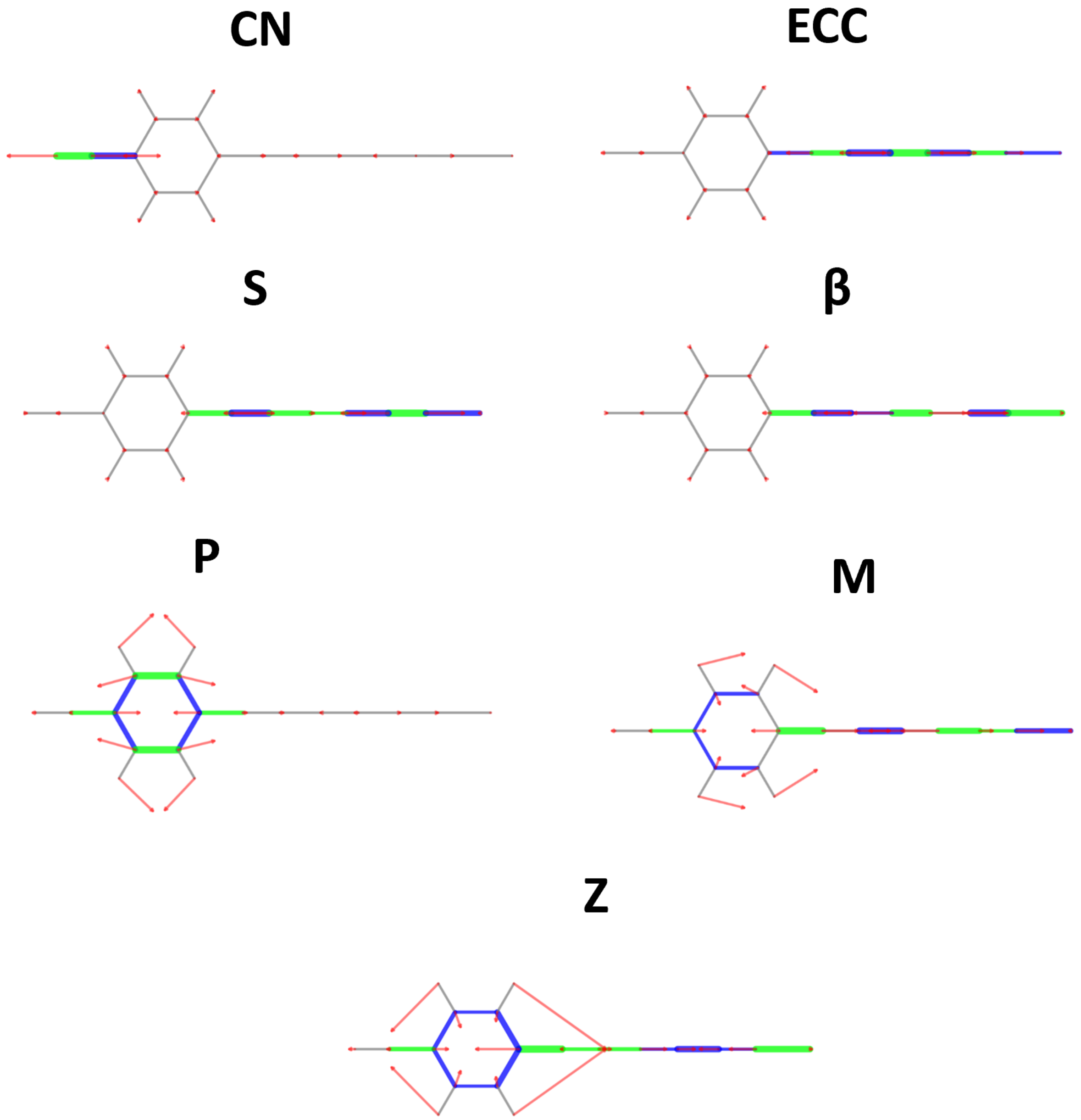

Figure S11 Graphical representation of the vibrational eigenvectors of the main normal modes of C_6Br molecule taken as a reference for the other 1-halopolyynes investigated in this work. Red arrows represent displacement vectors; CC bonds are represented as green (blue) lines of different thicknesses according to their relative stretching (shrinking).

ii. *Synthesis and characterization of new polyynes*

All reactions were carried out using standard Schlenk technique. MeCN was distilled over P_2O_5, other solvents were used without further purification. Following reagents were used without further purification: NCS (Fluorochem, 98%), NBS (Alfa Aesar, 99%), $AgNO_3$ (POCH, pure for analysis), AgF (Alfa Aesar, 98%), and TBAF (Sigma Aldrich, 1M in THF). Polyynes S1 and S2 were obtained according to the known procedures[1,2]. Chlorination and bromination procedures were adapted from the literature[1,3].

^{1}H and ^{13}C NMR spectra were recorded with a Bruker Avance 500 MHz spectrometer with an inverse broadband probe. For all the NMR spectra, the chemical shifts are given in ppm relative to the solvent residual peaks ($CDCl_3$, ^{1}H: 7.26 ppm, ^{13}C: 77.16 ppm). MS spectra were recorded using a Bruker qTOF spectrometer with an ESI ion source or a JOEL

Synthesis of C_6Cl

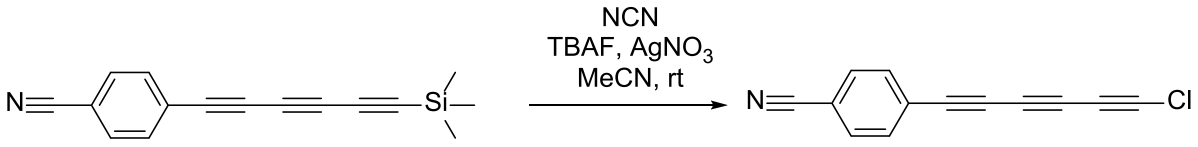

Polyyne **S1** (0.088 g, 0.36 mmol) was dissolved in a dry MeCN (10 mL) and placed nitrogen purged Schlenk flask. Next *N*-chlorosuccinimide (NCS, 0.079 g, 0.58 mmol) and $AgNO_3$ (0.015 g, 0.90 mmol) were added and white precipitate was formed. At the end TBAF (tetra-*n*-butylammonium fluoride, 1M in THF) was added dropwise and the reaction mixture was stirred for 18 h in an inert atmosphere. After completion of reaction the solvent was removed under reduced pressure and product was purified using short silica gel plug (hexane/CH_2Cl_2, *v*/*v*, 1/1) yielding C_6Cl as a white microcrystalline solid (0.060 g, 0.29 mmol). Yield: 80%

Caution! Solid C_6Cl decomposes at room temperature and might explode when exposed to heat! However, it might be stored in a freezer for weeks.

^{1}H NMR (500 MHz, $CDCl_3$) δ 7.64 – 7.58 (m, 4H).

^{13}C NMR (126 MHz, $CDCl_3$) δ 133.7, 132.3, 125.8, 118.2, 113.2, 78.3, 73.3, 68.8, 60.9, 58.4, 55.9.

HRMS (ESI): *m*/*z* calculated for $C_{13}H_5NCl$ $[M+H]^+$: 210.0105; measured: 210.0091.

Synthesis of C_8Br

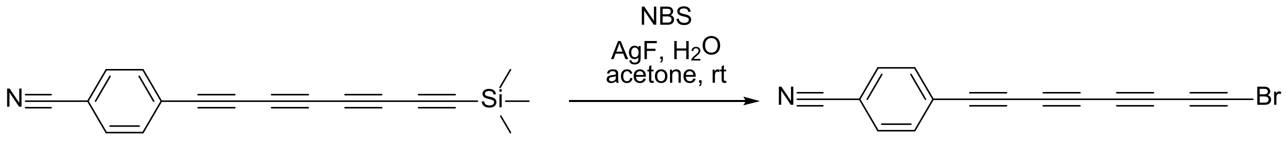

Polyyne **S2** (0.100 g, 0.368 mmol) was dissolved in an acetone (10 mL) and placed nitrogen purged Schlenk flask wrapped in aluminum foil. Next *N*-bromosuccinimide (NBS, 0.072 g, 0.41 mmol), AgF (0.014 g, 0.11 mmol) were added and the reaction mixture was stirred for 24 h in an inert atmosphere in a dark. After completion of reaction the solvent was removed under reduced pressure and product was purified using short silica gel plug (hexane/CH_2Cl_2, *v*/*v*, 1/3) yielding C_8Br as a yellow microcrystalline solid (0.043 g, 0.15 mmol). Yield: 41%.

Caution! Solid C_8Br rapidly decomposes at room temperature and might explode when exposed to heat! However, it might be stored in a freezer for days.

^{1}H NMR (500 MHz, $CDCl_3$) δ 7.65 – 7.59 (m, 4H).

^{13}C NMR (126 MHz, $CDCl_3$) δ 133.8, 132.3, 125.5, 118.1, 113.4, 78.3, 74.1, 69.4, 66.1, 65.8, 59.9, 59.3, 43.9.

MS (MALDI): *m*/*z* calculated for $C_{15}H_4N$ $[M-Br]^+$: 198.0344, measured: 198.0144.

NMR spectra

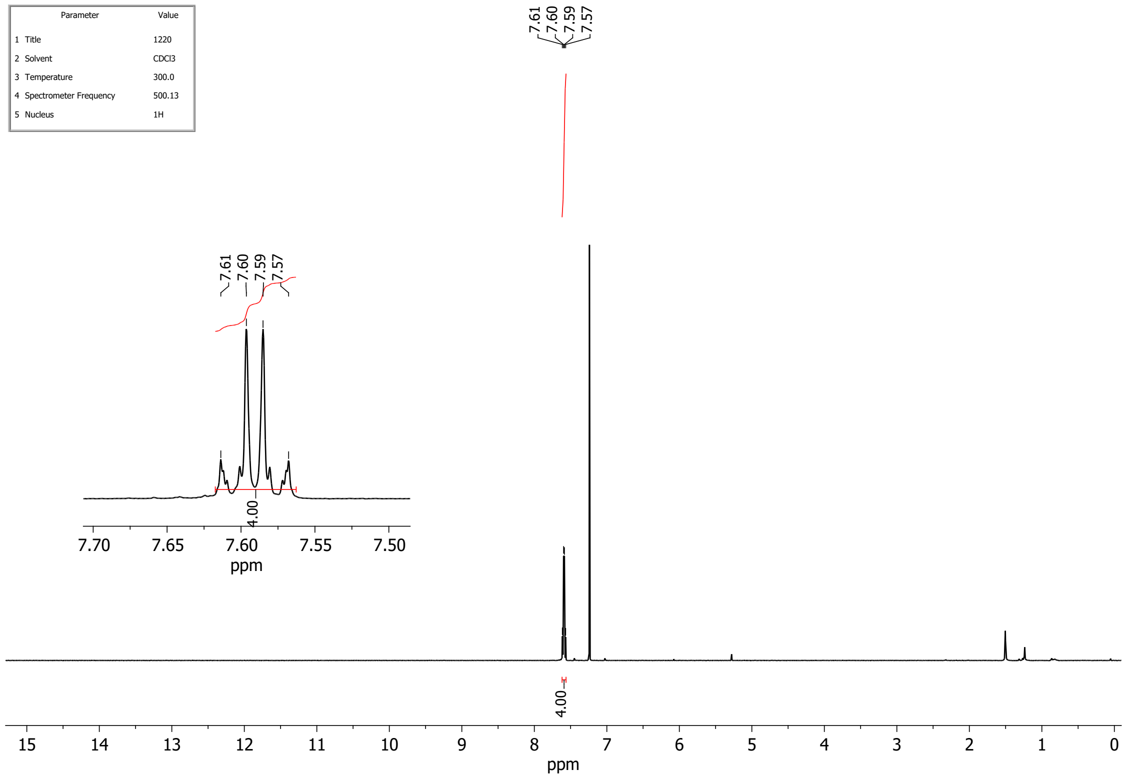

Figure S12. ^{1}H NMR spectrum of C_6Cl (500 MHz, 300 K, $CDCl_3$).

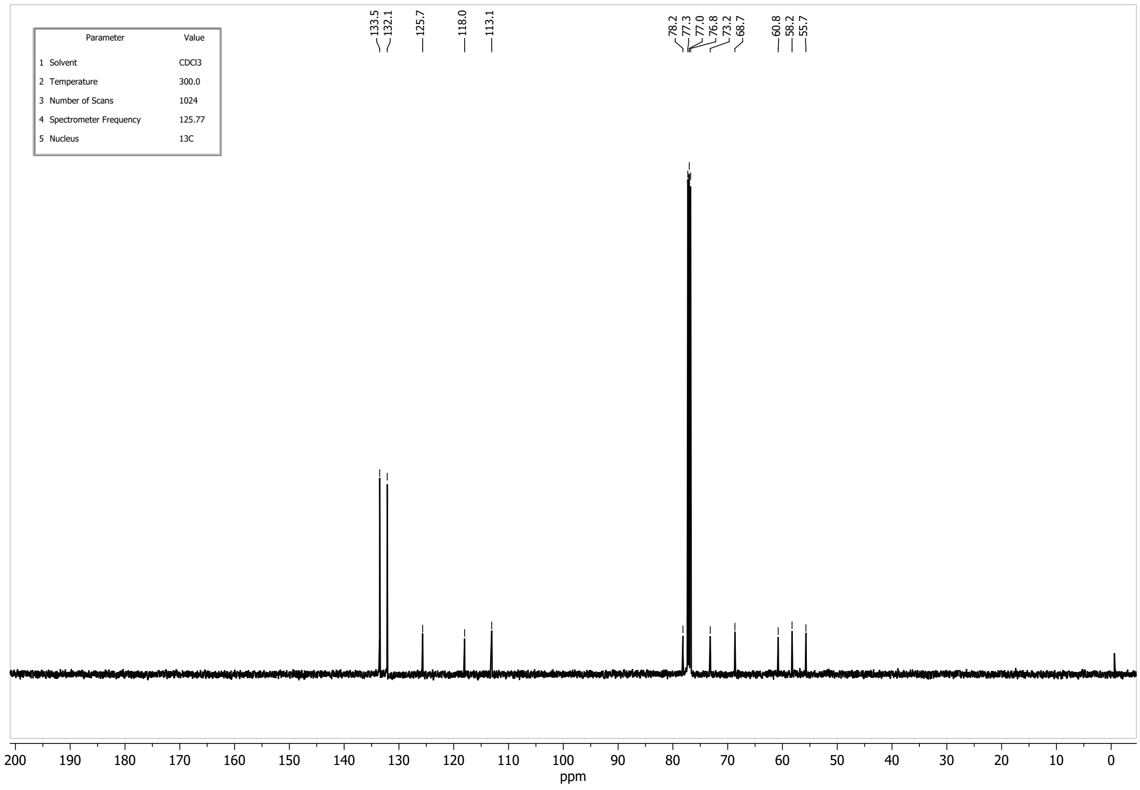

Figure S13. ^{13}C NMR spectrum of C_6Cl (126 MHz, 300 K, $CDCl_3$).

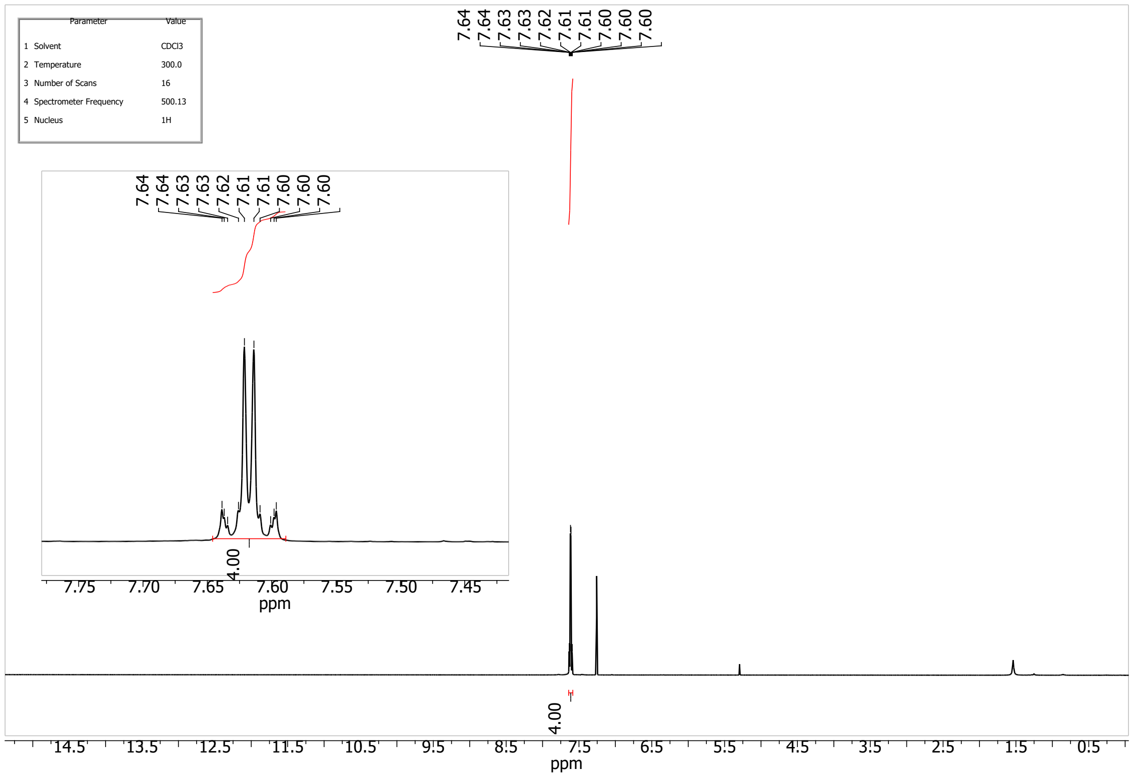

Figure S14. ^{1}H NMR spectrum of C_8Br (500 MHz, 300 K, $CDCl_3$).

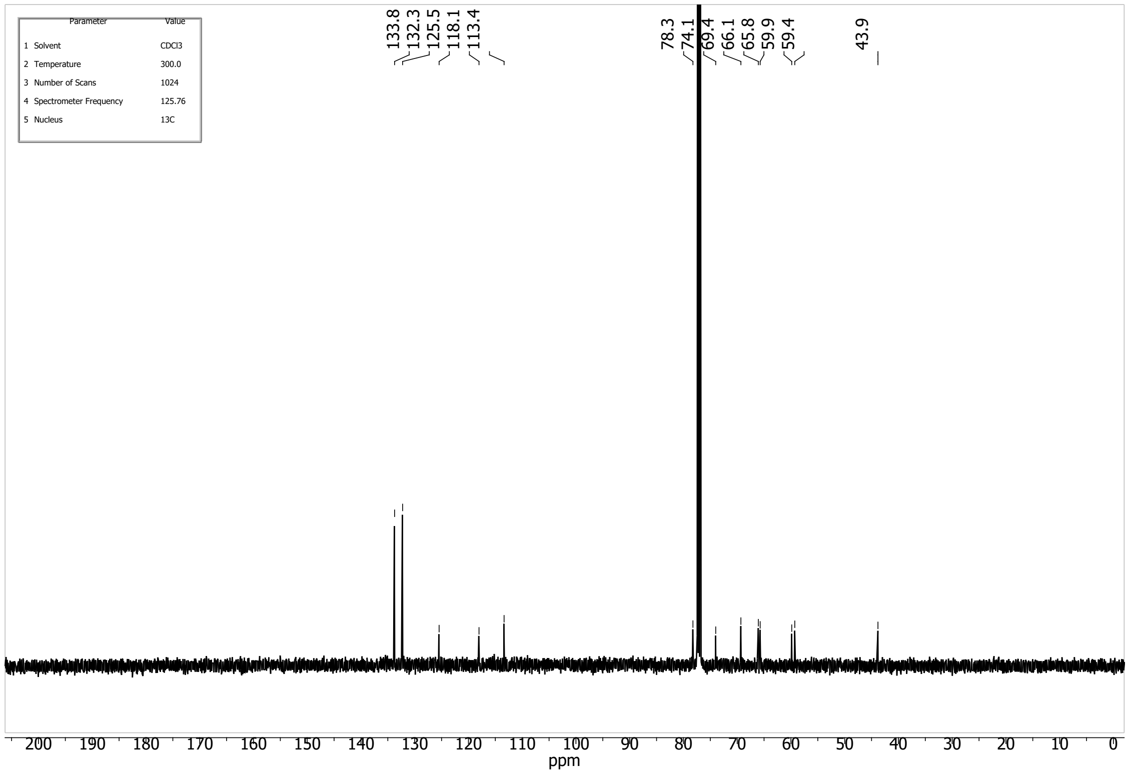

Figure S15. ^{13}C NMR spectrum of C_8Br (126 MHz, 300 K, $CDCl_3$).

iii. X-ray crystallography details

Table S4 Crystal data and structure refinement for C_4Cl.

Identification code	C_4Cl (CCDC 2281262)
Empirical formula	$C_{11}H_4ClN$
Formula weight	185.60
Temperature/K	100
Crystal system	triclinic
Space group	P-1
a/Å	3.8030(10)
b/Å	9.293(2)
c/Å	12.586(3)
α/°	81.99(3)
β/°	89.21(3)
γ/°	89.46(3)
Volume/Å^3	440.41(19)
Z	2
ρ_{calc}g/cm^3	1.400
μ/mm^{-1}	0.375
F(000)	188.0
Crystal size/mm^3	0.346 × 0.261 × 0.113
Radiation	Mo Kα (λ = 0.71073)
2Θ range for data collection/°	7.37 to 74.596
Index ranges	$-6 \leq h \leq 6$, $-15 \leq k \leq 15$, $-21 \leq l \leq 19$
Reflections collected	8869
Independent reflections	4289 [R_{int} = 0.0333, R_{sigma} = 0.0495]
Data/restraints/parameters	4289/0/118
Goodness-of-fit on F^2	1.143
Final R indexes [I>=2σ (I)]	R_1 = 0.0762, wR_2 = 0.2003
Final R indexes [all data]	R_1 = 0.0974, wR_2 = 0.2117
Largest diff. peak/hole / e Å^{-3}	0.86/-0.48

Table S5 Crystal data and structure refinement for C_4Br.

Identification code	C_4Br (CCDC 2281263)
Empirical formula	$C_{11}H_4BrN$
Formula weight	230.06
Temperature/K	100.00(10)
Crystal system	monoclinic
Space group	$P2_1/c$
a/Å	3.9000(10)
b/Å	16.613(4)
c/Å	14.212(3)
α/°	90
β/°	97.85(3)
γ/°	90
Volume/Å^3	912.2(4)
Z	4
ρ_{calc}g/cm^3	1.675
μ/mm^{-1}	5.685
F(000)	448.0
Crystal size/mm^3	0.4011 × 0.0489 × 0.0294
Radiation	Cu Kα (λ = 1.54184)
2Θ range for data collection/°	8.232 to 150.976
Index ranges	-4 ≤ h ≤ 4, -19 ≤ k ≤ 20, -17 ≤ l ≤ 16
Reflections collected	6909
Independent reflections	1846 [R_{int} = 0.0192, R_{sigma} = 0.0140]
Data/restraints/parameters	1846/0/118
Goodness-of-fit on F^2	1.106
Final R indexes [I>=2σ (I)]	R_1 = 0.0302, wR_2 = 0.0802
Final R indexes [all data]	R_1 = 0.0304, wR_2 = 0.0803
Largest diff. peak/hole / e Å^{-3}	1.10/-0.29

Table S6 Crystal data and structure refinement for C_6Cl.

Identification code	C_6Cl (CCDC 2281261)
Empirical formula	$C_{13}H_4ClN$
Formula weight	209.62
Temperature/K	100.15
Crystal system	monoclinic
Space group	$P2_1/c$
a/Å	3.7672(10)
b/Å	25.323(6)
c/Å	10.466(3)
α/°	90
β/°	94.84(2)
γ/°	90
Volume/Å^3	994.8(5)
Z	4
ρ_{calc}g/cm^3	1.400
μ/mm^{-1}	3.050
F(000)	424.0
Crystal size/mm^3	0.155 × 0.098 × 0.057
Radiation	CuKα (λ = 1.54184)
2Θ range for data collection/°	6.982 to 146.438
Index ranges	$-4 \leq h \leq 4$, $-30 \leq k \leq 29$, $-12 \leq l \leq 12$
Reflections collected	4608
Independent reflections	1919 [R_{int} = 0.0082, R_{sigma} = 0.0094]
Data/restraints/parameters	1919/0/136
Goodness-of-fit on F^2	1.077
Final R indexes [I>=2σ (I)]	R_1 = 0.0368, wR_2 = 0.0987
Final R indexes [all data]	R_1 = 0.0383, wR_2 = 0.0994
Largest diff. peak/hole / e Å^{-3}	0.30/-0.34

Table S7 Crystal data and structure refinement for C_6Br.

Identification code	C_6Br (CCDC 2281264)
Empirical formula	$C_{13}H_4NBr$
Formula weight	254.08
Temperature/K	99.98(10)
Crystal system	monoclinic
Space group	$P2_1/c$
a/Å	3.83030(10)
b/Å	25.7861(5)
c/Å	10.3989(2)
α/°	90
β/°	94.395(2)
γ/°	90
Volume/Å^3	1024.06(4)
Z	4
ρ_{calc}g/cm^3	1.648
μ/mm^{-1}	5.134
F(000)	496.0
Crystal size/mm^3	0.181 × 0.044 × 0.019
Radiation	CuKα (λ = 1.54184)
2Θ range for data collection/°	6.856 to 146.036
Index ranges	$-4 \le h \le 4$, $-31 \le k \le 29$, $-12 \le l \le 11$
Reflections collected	11377
Independent reflections	2013 [R_{int} = 0.0247, R_{sigma} = 0.0170]
Data/restraints/parameters	2013/0/136
Goodness-of-fit on F^2	1.059
Final R indexes [I>=2σ (I)]	R_1 = 0.0271, wR_2 = 0.0741
Final R indexes [all data]	R_1 = 0.0291, wR_2 = 0.0753
Largest diff. peak/hole / e Å^{-3}	0.89/-0.55

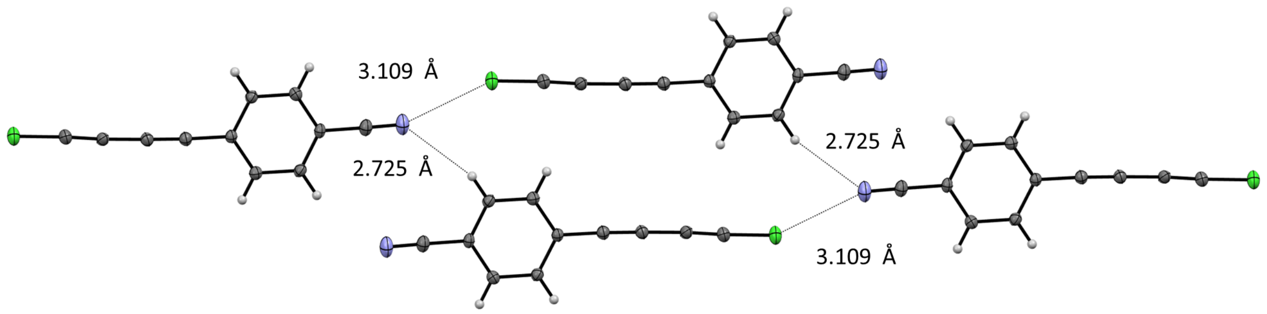

Figure S16 Crystal packing of C_4Cl. All thermal ellipsoids are given with a 50% probability level.

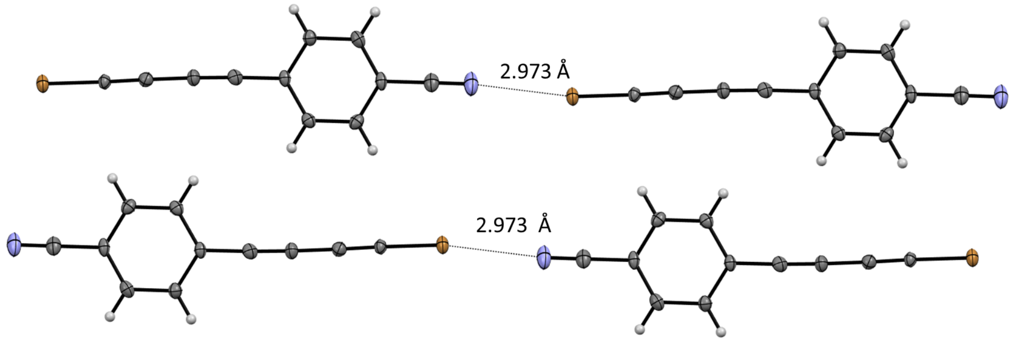

Figure S17 Crystal packing of C_4Br. All thermal ellipsoids are given with a 50% probability level.

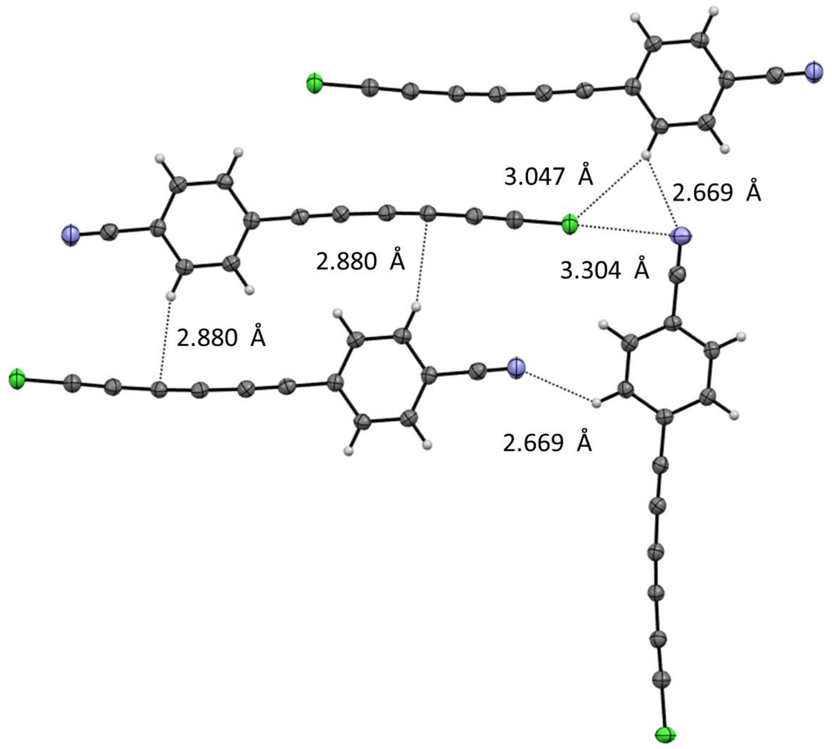

Figure S18 Crystal packing of C_6Cl. All thermal ellipsoids are given with a 50% probability level.

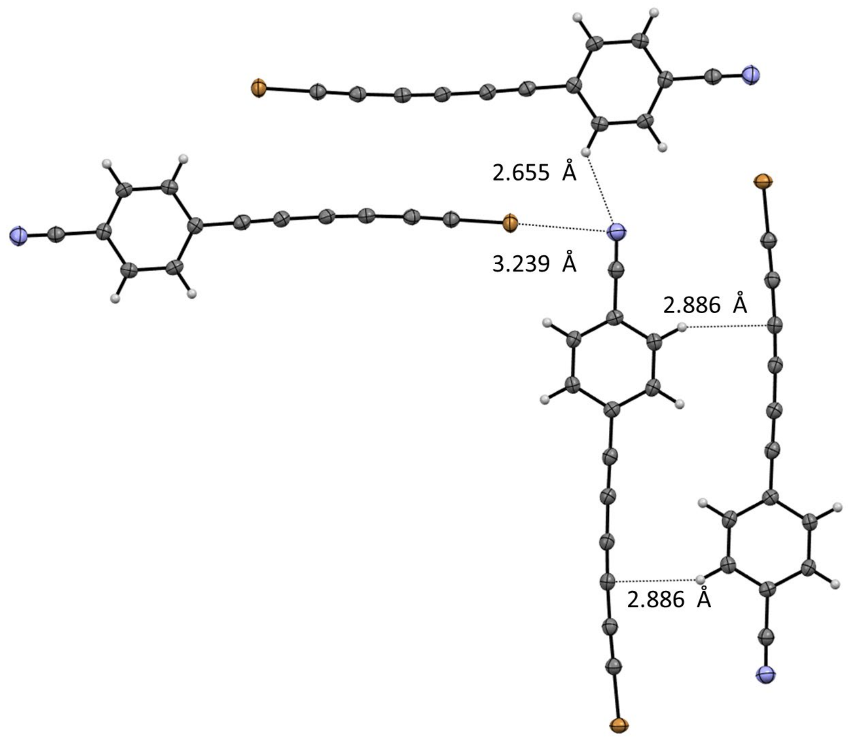

Figure S19 Crystal packing of C_6Br. All thermal ellipsoids are given with a 50% probability level.

Table S8. X…N contacts in solid-state structures of 1-halopolyynes and the values of the van der Waals radii used to calculate the ΔR/R values.

Compound	X…N / Å	∠(CXN) / deg	ΔR/R
C_4Cl^a	3.109	152.5	5.8%
C_4Br^a	2.973	163.8	12.5%
C_4I^b	2.883	177.0	18.3%
C_6Cl^a	3.239	176.6	1.8%
C_6Br^a	3.304	176.7	2.8%
C_6I^b	2.881	178.1	18.4%
C_8I^b	2.888	178.4	18.2%

	R_{Vdw}(X)/pm
Cl	175
Br	185
I	198
N	155

[a] This work

[b] Known structures from Pigulski et al.[4]

iv. *I*nteraction energies and Hirshfeld surface analysis

C4I

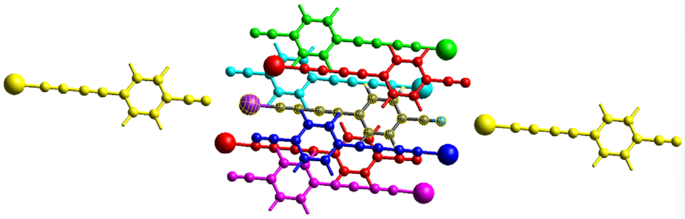

	N	Symop	R	E_ele	E_pol	E_dis	E_rep	E_tot
	0	x, y, z	4.05	-17.3	-1.0	-44.6	57.1	-22.6
	0	x, y, z	15.46	-34.2	-5.6	-3.6	0.0	-43.5
	0	-x, -y, -z	6.40	-14.3	-2.8	-28.7	30.4	-23.4
	0	-x, -y, -z	5.20	-15.0	-1.3	-33.2	34.7	-24.3
	0	-x, -y, -z	6.54	-15.0	-2.1	-34.3	34.6	-26.0
	0	-x, -y, -z	6.00	-14.7	-2.7	-30.4	32.1	-24.2

C6I

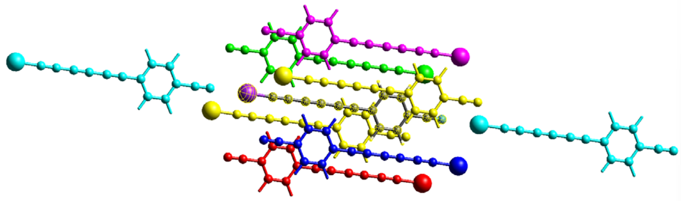

	N	Symop	R	Electron Density	E_ele	E_pol	E_dis	E_rep	E_tot
	1	-x, -y, -z	6.25	B3LYP/DGDZVP	-15.4	-2.8	-30.6	32.6	-25.0
	2	x, y, z	4.08	B3LYP/DGDZVP	-19.0	-1.2	-50.0	63.1	-25.5
	1	-x, -y, -z	5.61	B3LYP/DGDZVP	-16.8	-1.5	-37.7	38.0	-28.2
	2	x, y, z	18.02	B3LYP/DGDZVP	-31.6	-5.7	-3.6	0.0	-40.7
	1	-x, -y, -z	6.90	B3LYP/DGDZVP	-16.9	-2.0	-35.9	37.0	-27.8
	1	-x, -y, -z	7.04	B3LYP/DGDZVP	-15.9	-3.1	-30.3	32.0	-25.7

C8I

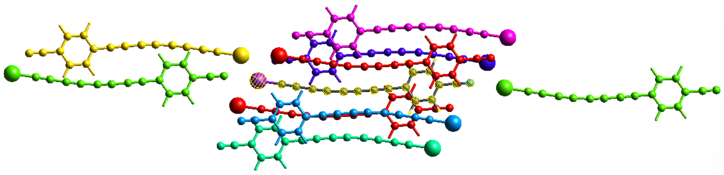

	N	Symop	R	Electron Density	E_ele	E_pol	E_dis	E_rep	E_tot
	2	x, y, z	4.05	B3LYP/DGDZVP	-21.4	-1.2	-56.4	72.5	-27.9
	1	-x, -y, -z	14.98	B3LYP/DGDZVP	-1.4	-0.2	-6.5	0.0	-7.3
	2	x, y, z	20.48	B3LYP/DGDZVP	-26.7	-5.5	-3.6	0.0	-35.5
	1	-x, -y, -z	5.21	B3LYP/DGDZVP	-13.7	-2.5	-35.7	43.0	-20.9
	1	-x, -y, -z	5.57	B3LYP/DGDZVP	-16.6	-1.5	-42.9	45.1	-28.2
	1	-x, -y, -z	7.29	B3LYP/DGDZVP	-18.7	-2.7	-39.6	41.4	-30.7
	1	-x, -y, -z	8.90	B3LYP/DGDZVP	-12.3	-3.7	-30.9	27.8	-25.5

C4Br

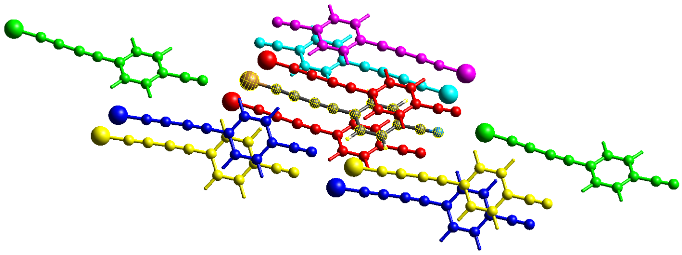

	N	Symop	R	Electron Density	E_ele	E_pol	E_dis	E_rep	E_tot
	2	x, y, z	3.90	B3LYP/DGDZVP	-14.4	-1.0	-45.5	52.8	-23.0
	2	x, -y+1/2, z+1/2	9.60	B3LYP/DGDZVP	-5.5	-1.3	-13.5	16.3	-8.5
	2	x, y, z	15.24	B3LYP/DGDZVP	-20.3	-2.8	-3.2	0.0	-26.3
	1	-x, -y, -z	5.09	B3LYP/DGDZVP	-13.3	-2.5	-27.9	28.8	-22.4
	2	x, -y+1/2, z+1/2	8.33	B3LYP/DGDZVP	-11.7	-1.6	-15.8	14.3	-18.5
	1	-x, -y, -z	5.31	B3LYP/DGDZVP	-14.5	-2.1	-32.7	32.0	-25.6

C6Br

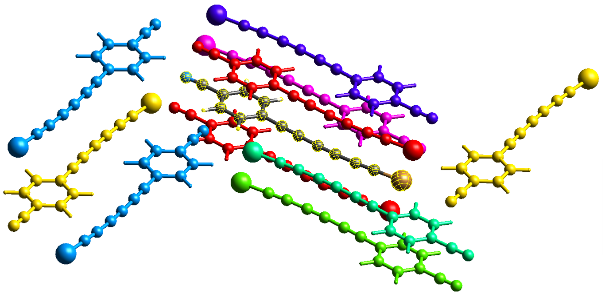

	N	Symop	R	E_ele	E_pol	E_dis	E_rep	E_tot
	0	x, y, z	3.83	-17.0	-1.1	-52.3	63.1	-25.4
	0	-x, y+1/2, -z+1/2	12.89	-17.6	-1.8	-9.1	0.0	-27.9
	0	-x, -y, -z	5.32	-10.0	-0.7	-29.7	35.6	-14.9
	0	-x, -y, -z	6.26	0.7	-1.1	-22.8	22.8	-5.9
	0	x, -y+1/2, z+1/2	13.92	-5.6	-3.0	-6.0	0.0	-13.3
	0	-x, -y, -z	5.12	-14.7	-1.8	-32.5	30.1	-26.6
	0	-x, -y, -z	6.21	-13.4	-3.0	-28.1	26.9	-24.3

Figure S20 Interaction energies (E_tot. in kJ/mol) of adjacent molecules calculated using *CrystalExplorer* (B3LYP/DGDZVP).

C4Cl

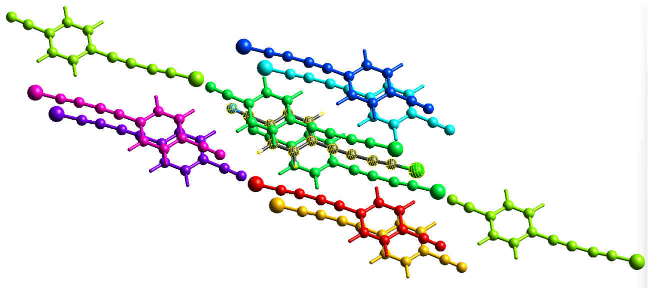

	N	Symop	R	Electron Density	E_ele	E_pol	E_dis	E_rep	E_tot
	1	-x, -y, -z	5.75	B3LYP/DGDZVP	-8.1	-1.5	-21.4	25.5	-12.5
	1	-x, -y, -z	6.07	B3LYP/DGDZVP	-4.6	-0.6	-19.5	18.7	-10.7
	2	x, y, z	15.08	B3LYP/DGDZVP	-12.4	-1.3	-2.9	0.0	-16.6
	2	x, y, z	3.80	B3LYP/DGDZVP	-12.7	-0.9	-44.3	51.0	-21.2
	1	-x, -y, -z	4.82	B3LYP/DGDZVP	-11.8	-2.6	-25.7	27.4	-19.9
	1	-x, -y, -z	4.32	B3LYP/DGDZVP	-14.6	-2.0	-32.4	32.4	-25.2
	1	-x, -y, -z	11.40	B3LYP/DGDZVP	-10.8	-2.7	-11.6	13.3	-15.3
	1	-x, -y, -z	12.47	B3LYP/DGDZVP	-18.6	-3.7	-8.6	0.0	-29.9

C6Cl

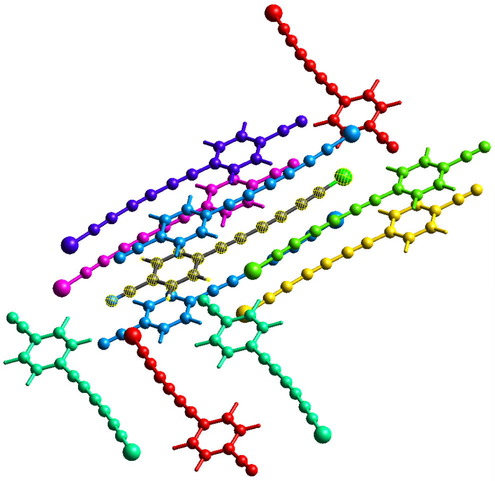

	N	Symop	R	Electron Density	E_ele	E_pol	E_dis	E_rep	E_tot
	2	-x, y+1/2, -z+1/2	12.78	B3LYP/DGDZVP	-0.7	-1.1	-7.0	0.0	-7.7
	1	-x, -y, -z	7.19	B3LYP/DGDZVP	-7.7	-0.6	-25.5	30.2	-12.2
	1	-x, -y, -z	8.17	B3LYP/DGDZVP	-1.5	-0.9	-21.0	22.6	-6.6
	2	x, -y+1/2, z+1/2	12.21	B3LYP/DGDZVP	-9.0	-2.9	-6.0	0.0	-17.0
	2	x, y, z	3.77	B3LYP/DGDZVP	-15.8	-1.1	-51.3	61.5	-24.2
	1	-x, -y, -z	4.27	B3LYP/DGDZVP	-15.5	-1.9	-32.6	31.5	-26.7
	1	-x, -y, -z	4.98	B3LYP/DGDZVP	-13.0	-3.2	-27.7	27.3	-23.3

Figure S21 Interaction energies (E_tot. in kJ/mol) of adjacent molecules calculated using *CrystalExplorer* (B3LYP/DGDZVP).

Table S9 Types of contacts on Hirshfeld surfaces calculated using *CrystalExplorer*.

**C4I**	Total Hirshfeld surface: 248.76 Å^2	
Interactions	Surface (%)	Surface (Å^2)
I...N / N...I	20.20%	50.25
C...HC / CH...C	44.80%	111.44
C...C	11.30%	28.11
**C6I**	Total Hirshfeld surface 281.77 Å^2	
Interactions	Surface (%)	Surface (Å^2)
I...N / N...I	18.80%	52.97
C...HC / CH...C	49.70%	140.04
C...C	15.20%	42.83
**C8I**	Total Hirshfeld surface 311.86 Å^2	
Interactions	Surface (%)	Surface (Å^2)
I...N / N...I	11.20%	34.93
C...HC / CH...C	36.90%	115.08
C...C	27.50%	85.76
**C4Br**	Total Hirshfeld surface 235.39 Å^2	
Interactions	Surface (%)	Surface (Å^2)
Br...N / N...Br	12.30%	28.95
C...HC / CH...C	40.60%	95.57
C...C	14.90%	35.07
**C6Br**	Total Hirshfeld surface: 266.48 Å^2	
Interactions	Surface (%)	Surface (Å^2)
Br...N / N...Br	3.90%	10.39
C...HC / CH...C	29.60%	78.88
C...C	27.50%	73.28
**C4Cl**	Total Hirshfeld surface: 230.32 Å^2	
Interactions	Surface (%)	Surface (Å^2)
Cl...N / N...Cl	8.80%	20.27
C...HC / CH...C	38.60%	90.86
C...C	17.80%	41.90
**C6Cl**	Total Hirshfeld surface: 260.87 Å^2	
Interactions	Surface (%)	Surface (Å^2)
Cl...N / N...Cl	3.50%	9.13
C...HC / CH...C	27.90%	72.78
C...C	28.80%	75.13

C4I

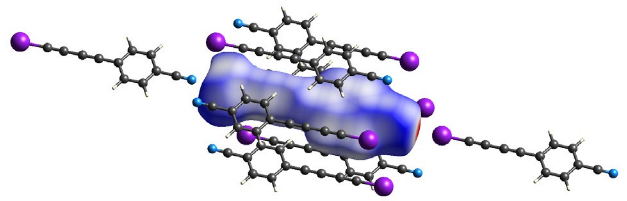

C6I

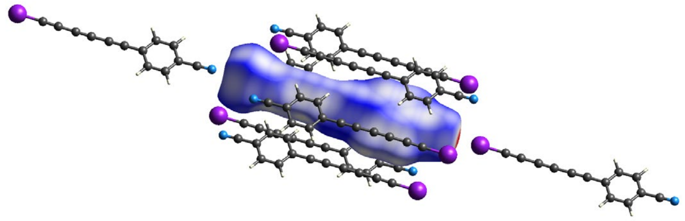

C8I

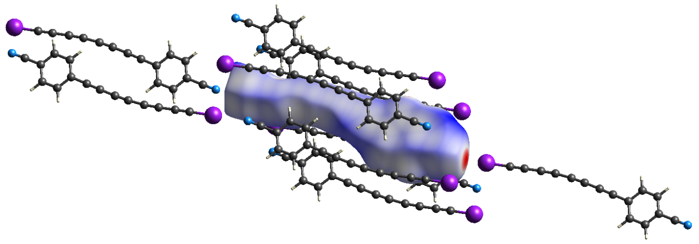

C4Br

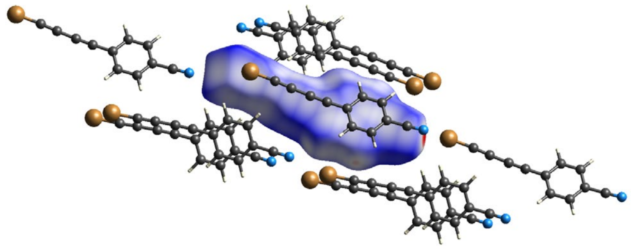

Figure S22 Hirshfeld surfaces of C4I, C6I, C8I, and C4Br calculated using *CrystalExplorer*.

C6Br

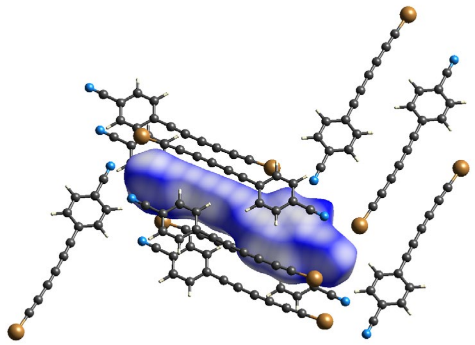

C4Cl

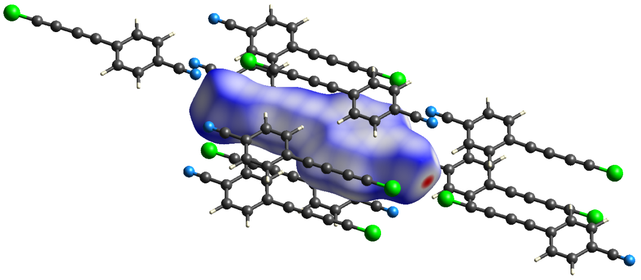

C6Cl

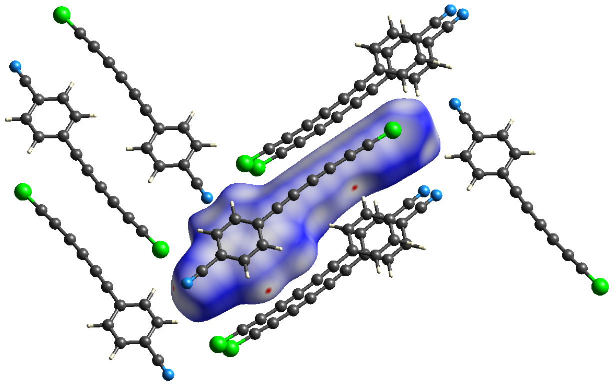

Figure S23 Hirshfeld surfaces of C6Br, C4Cl, and C6Cl calculated using *CrystalExplorer*.

v. References

(1) Gulia, N.; Pigulski, B.; Szafert, S. Palladium End-Capped Polyynes via Oxidative Addition of 1 - Haloalkynes to Pd(PPh3)4. *Organometallics* **2015**, *34*, 673–682. https://doi.org/10.1021/om501105w.

(2) Pigulski, B.; Gulia, N.; Szafert, S. Synthesis of Long , Palladium End-Capped Polyynes through the Use of Asymmetric 1-Iodopolyynes. *Chemistry Europe Journal* **2015**, *21*, 17769–17778. https://doi.org/10.1002/chem.201502737.

(3) Gulia, N.; Pigulski, B.; Charewicz, M.; Szafert, S. A Versatile and Highly Efficient Method for 1-Chlorination of Terminal and Trialkylsilyl-Protected Alkynes. *Chemistry–A European Journal* **2014**, *20* (10), 2746–2749.

(4) Pigulski, B.; Gulia, N.; Męcik, P.; Wieczorek, R.; Arendt, A.; Szafert, S. Crystal Engineering of 1-Halopolyynes by End-Group Manipulation. *Cryst Growth Des* **2019**, *19* (11), 6542–6551. https://doi.org/10.1021/acs.cgd.9b00987.
